# Synaptic Dynamics Convey Differential Sensitivity to Input Pattern Changes in Two Muscles Innervated by the Same Motor Neurons

**DOI:** 10.1523/ENEURO.0351-21.2021

**Published:** 2021-11-19

**Authors:** Nelly Daur, Farzan Nadim, Dirk Bucher

**Affiliations:** 1 Federated Department of Biological Sciences, New Jersey Institute of Technology and Rutgers University-Newark, Newark, NJ 07102; 2 Department of Mathematical Sciences, New Jersey Institute of Technology, Newark, NJ 07102

**Keywords:** bursting neuron, depression, facilitation, frequency filtering, short-term synaptic plasticity, stomatogastric

## Abstract

Postsynaptic responses depend on input patterns as well as short-term synaptic plasticity, summation, and postsynaptic membrane properties, but the interactions of those dynamics with realistic input patterns are not well understood. We recorded the responses of the two pyloric dilator (PD) muscles, *cpv2a* and *cpv2b*, that are innervated by and receive identical periodic bursting input from the same two motor neurons in the lobster *Homarus americanus*. *Cpv2a* and *cpv2b* showed quantitative differences in membrane nonlinearities and synaptic summation. At a short timescale, responses in both muscles were dominated by facilitation, albeit with different frequency and time dependence. Realistic burst stimulations revealed more substantial differences. Across bursts, *cpv2a* showed transient depression, whereas *cpv2b* showed transient facilitation. Steady-state responses to bursting input also differed substantially. Neither muscle had a monotonic dependence on frequency, but *cpv2b* showed particularly pronounced bandpass filtering. *Cpv2a* was sensitive to changes in both burst frequency and intra-burst spike frequency, whereas, despite its much slower responses, *cpv2b* was largely insensitive to changes in burst frequency. *Cpv2a* was sensitive to both burst duration and number of spikes per burst, whereas *cpv2b* was sensitive only to the former parameter. Neither muscle showed consistent sensitivity to changes in the overall spike interval structure, but *cpv2b* was surprisingly sensitive to changes in the first intervals in each burst, a parameter known to be regulated by dopamine (DA) modulation of spike propagation of the presynaptic axon. These findings highlight how seemingly minor circuit output changes mediated by neuromodulation could be read out differentially at the two synapses.

## Visual Abstract

**Figure:**
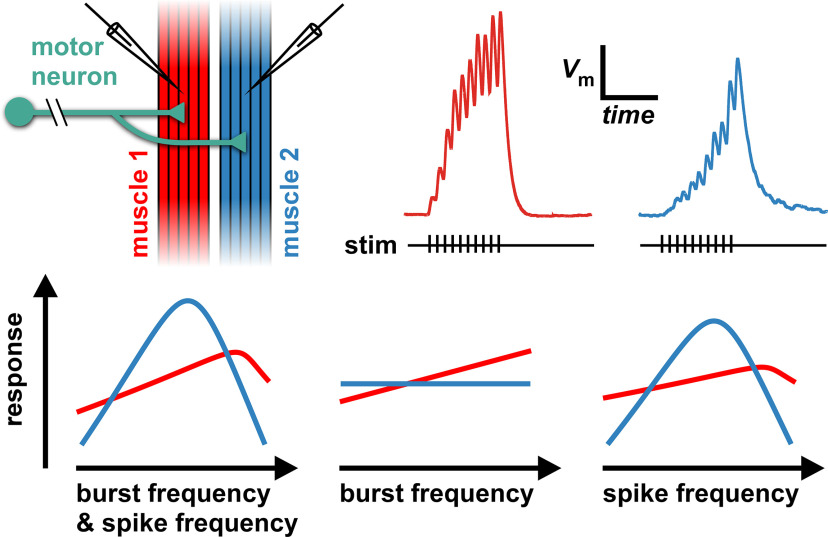

## Significance Statement

Studies of neural coding have focused mainly on patterns of activity, and less on how postsynaptic targets read out these inputs, which is dependent on short-term synaptic plasticity and postsynaptic membrane properties. We show that subthreshold postsynaptic responses at two crustacean neuromuscular junctions (NMJs) formed by the same motor neuron increase in strength with repeated activation. However, stimulation with more complex bursting patterns that mimicked different circuit states revealed very different sensitivities to specific pattern attributes. Consequently, presynaptic and postsynaptic dynamics enabled differential readout of input changes arising from circuit modulation. Such differential readout may play an important role in neural processing in general, as different pathways may pay attention to different information encoded in the same activity patterns.

## Introduction

Neural circuits can produce a wide range of different activity patterns, dependent on behavioral context and internal state. Activity varies with sensory inputs, neuromodulation, and long-term plasticity, as circuit activation, intrinsic neuronal excitability, and synaptic properties change (Briggman and Kristan, 2008; Nadim and Bucher, 2014; Hahn et al., 2019; McCormick et al., 2020). Even under stable conditions, some features of neuronal activity can be highly variable, while others are well preserved, both within and across individuals. For example, across-trial variability of cortical activity differs over the time course of sensory stimulation, independent of mean responses (Churchland et al., 2010). In the crustacean stomatogastric ganglion (STG), the relative timing across different bursting neurons is well preserved across individuals, while burst frequency and intra-burst spike frequencies are not (Bucher et al., 2005). The functional significance of changes or variability in circuit output are often taken at face value, but the degree to which they lead to modifications in behavior depends on how well they can be discriminated by downstream readout (Stringer et al., 2021). An important part of this readout is the dynamics of postsynaptic responses, as both postsynaptic membrane nonlinearities and synaptic plasticity at different timescales determine which features of temporal patterns (and changes thereof) have a substantial impact on postsynaptic responses, and which do not (Hutcheon and Yarom, 2000; Fortune and Rose, 2001; Abbott and Regehr, 2004; Panzeri et al., 2010; Ainsworth et al., 2012; Regehr, 2012; Tseng et al., 2014; Anwar et al., 2017; Fox et al., 2017).

In motor systems, assessment of circuit activity is relatively straightforward, as such circuits produce an unambiguous neural output that can be measured as the patterns of action potentials that activate muscles. Rhythmic motor activity driven by central pattern generators (CPGs), in particular, can be recorded in isolated nervous system preparations, and readily defines lower motor neuron output in the absence of confounding sensory feedback (Marder and Bucher, 2001; Grillner, 2006; Bucher et al., 2015). Oscillatory activity also allows for straightforward quantification of output attributes in time, frequency, and phasing across neurons that are malleable to changes in circuit activation, for example by neuromodulators (Dickinson, 2006; Doi and Ramirez, 2008; Harris-Warrick, 2011; Miles and Sillar, 2011; Marder, 2012; Nusbaum and Blitz, 2012; Bucher and Marder, 2013; Diaz-Ríos et al., 2017; Ramirez and Baertsch, 2018).

To better understand the degree to which postsynaptic readout can differentiate between different attributes that characterize rhythmic motor neuron input, we studied the electrical responses of two crustacean stomach muscles that are innervated by the same motor neurons. The CPGs of the crustacean stomatogastric nervous system (STNS) that control stomach movements have been on the forefront of the study of circuit dynamics and neuromodulation (Marder and Bucher, 2007; Daur et al., 2016). In the STG, a plethora of neuromodulators tune neuronal and synaptic properties to generate circuit outputs that differ in attributes like burst frequencies, burst durations, intra-burst spike frequencies, and relative timing (Nusbaum et al., 2001; Dickinson, 2006; Marder and Bucher, 2007; Stein, 2009; Harris-Warrick, 2011). The functional significance of such changes for muscle activation are not well understood. Neuromuscular junctions (NMJs) at the striated stomatogastric muscles show diverse synaptic dynamics (Jorge-Rivera et al., 1998; Stein et al., 2006; Blitz et al., 2017), and muscle contractions are shaped by frequency filtering of neural input (Hooper and Weaver, 2000). The two NMJs studied here both showed typical synaptic facilitation when probed with standard protocols to assess synaptic dynamics, but substantial differences in responses to realistic bursting inputs. Our findings highlight that even without obvious qualitative differences in synaptic dynamics, postsynaptic responses can differ substantially in their sensitivity to different input attributes.

## Materials and Methods

### Experimental preparation

All experiments were performed on adult (∼500 g) lobsters, *Homarus americanus*, of either sex. Animals were obtained from Yankee Lobster Co in Boston, MA, or from local seafood stores in Newark, NJ, and kept unfed in tanks at 10–13°C. Before dissection, animals were cold-anesthetized in ice for 30–40 min. Reduced preparations of parts of the stomach and nerves were pinned into transparent Sylgard-lined (Dow-Corning) experimental dishes in physiological saline. Saline composition was as follows: 479.12 mm NaCl, 12.74 mm KCl, 13.67 mm CaCl_2_, 10 mm MgSO_4_, 3.91 mm Na_2_SO_4_, and 10 mm HEPES. The pH was adjusted to 7.4 –7.5.

**Figure 1.**
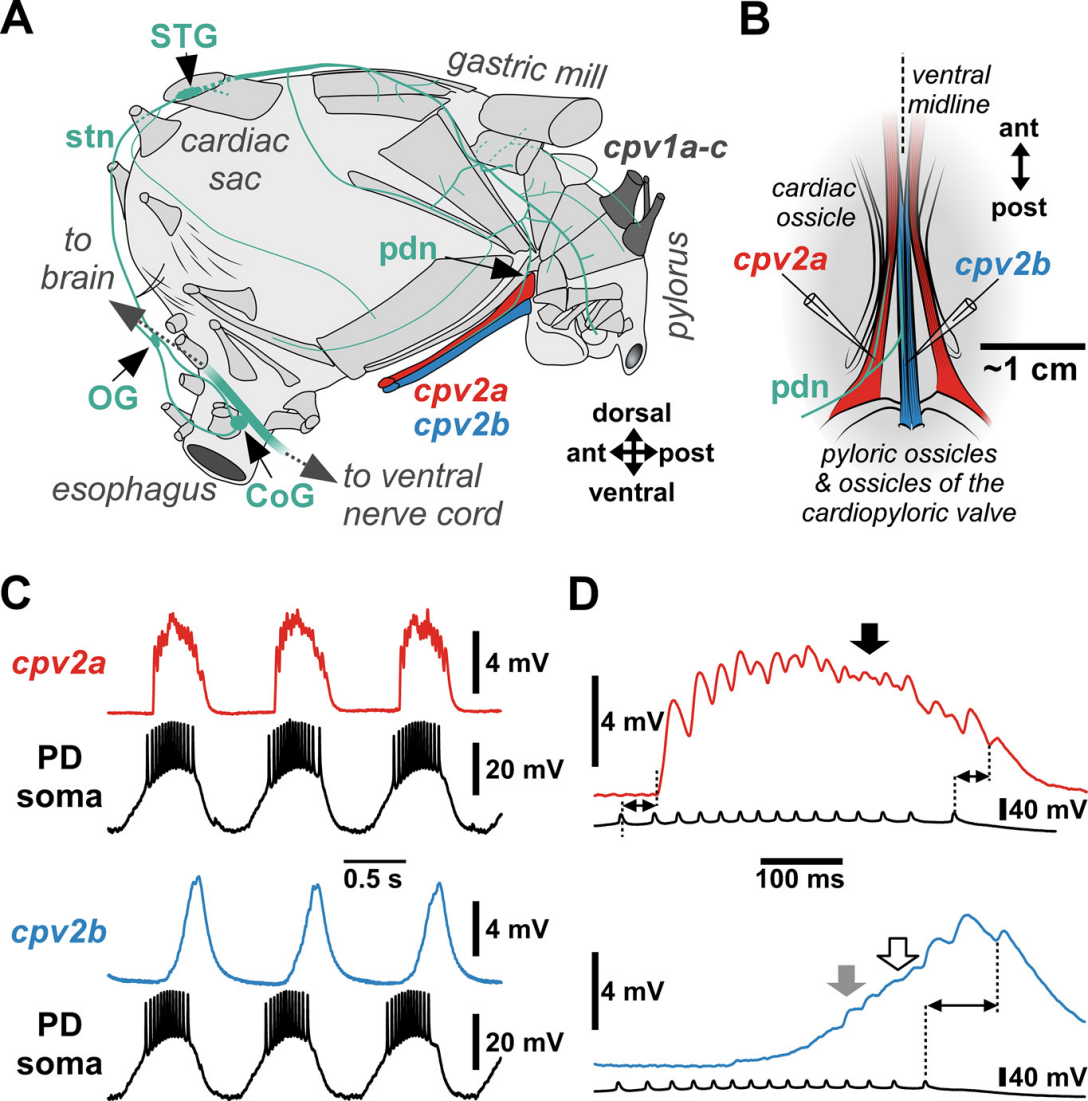
Intracellular recordings from ventral dilator muscles *cpv2a* and *cpv2b*. ***A***, Schematic side view of the *H. americanus* foregut. The nervous system is shown in teal, and the musculature in middle gray, except for dorsal dilators (dark gray) and ventral dilators (red and blue). Modified from Bucher et al. (2006) and Maynard and Dando (1974). STG, stomatogastric ganglion; OG, esophageal ganglion; CoG, commissural ganglion; stn, stomatogastric nerve; pdn, PD nerve; *cpv1&2*, cardiopyloric valve muscles. ***B***, Schematic ventral view at the level of the posterior cardiac sac. Intracellular recordings of *cpv2a* and *cpv2b* were obtained close to the insertions at pyloric and cardiopyloric valve ossicles. ***C***, Simultaneous recordings of a PD soma in the STG and *cpv2* fibers during rhythmic pyloric activity. ***D***, Compound synaptic responses to a single PD burst, showing the complex summation pattern of inputs from both PD neurons in *cpv2a* (black arrow), and different phases in *cpv2b*, characterized by smooth summation (white arrow) and local peaks (gray arrow). Dashed lines and double-sided arrows indicate the delay between PD spike peak in the STG and onset of the single EJP response for the first and last spike in *cpv2a*, and the last spike in *cpv2b*.

In all experiments, we recorded the synaptic responses of the ventral pyloric dilator (PD) muscles, *cpv2a* and *cpv2b*. Figure 1*A* shows a schematic of the *H. americanus* stomach musculature and STNS, following the description and nomenclature of Maynard and Dando (1974). The posterior-most part of the stomach, the pylorus, is connected to the cardiac sac via the cardiopyloric valve. It is constricted by a complex set of intrinsic muscles (with both attachment points at stomach ossicles) and dilated by two sets of extrinsic muscles attached close to the dorsal and ventral midlines to ossicles of the cardiopyloric valve and anterior pylorus. The dorsal dilators (cardiopyloric valve muscles, *cpv1*; Fig. 1*A*, dark gray) connect to carapace apodemes. The larger and more lateral ventral dilator (*cpv2a*; Fig. 1*A*,*B*, red) connects to the back of the esophagus, the smaller and more medial ventral dilator *(cpv2b*; Fig. 1*A*,*B*, blue) to the mandibles. Dorsal and ventral dilators are innervated by two PD neurons, which have their somata in the STG. The STG receives descending modulatory input from the paired commissural ganglia (CoGs) and the unpaired esophageal ganglion (OG) via the unpaired stomatogastric nerve (stn). This modulatory input activates CPG circuits in the STG that control the muscles of the gastric mill and pylorus.

The two PD neurons are part of the pyloric pacemaker kernel and are not bilateral homologues. Each has a single axon that leaves the STG and then splits once to project to both sides of the stomach, and a second time on each side to innervate the dorsal and ventral dilators. Therefore, each dilator muscle is innervated by both PD neurons. The ventral axon branches are carried by the pyloric dilator nerve (pdn), which innervates *cpv2a* and *cpv2b*.

Figure 1*B* shows a schematic of the experimental preparation. Both ventral dilator muscles consist of several centimeters long parallel fibers. At their posterior ends, they are attached to different ossicles, but converge to form a single bundle for most of their anterior path. The muscles were kept attached to their stomach ossicles as well as the esophageal and mandibular attachment sites. The underlying stomach lining was kept intact for stability and to pin the preparation ventral side up into the Sylgard dish without injuring the muscle. The ensheathing connective tissue was removed at the posterior part to allow intracellular muscle fiber recordings in the region where both muscles are clearly separated. Most of the rest of the stomach was removed. At least one of the pdns was cut proximal enough to allow building a petroleum jelly well around the cut end. The well served as electrical insulation for extracellular axon stimulation.

### Electrophysiology

During all recordings, preparations were superfused with saline cooled to 11–13°C by a custom-made Peltier device. For intracellular muscle fiber recordings, sharp glass electrodes were pulled with a Flaming-Brown P-97 puller (Sutter Instruments) and filled with 3 m KCl, yielding tip resistances of 15–25 MΩ. In some initial experiments, the STNS was kept largely intact along with the *cpv2* muscles, and PD neuron somata were recorded simultaneously with the muscle fibers. The STG was mechanically desheathed. Soma recordings were performed with electrodes filled with 0.6 m K_2_SO_4_ and 20 mm KCl to minimize alteration of chloride conductances. These electrodes yielded tip resistances of 20–30 MΩ. Signals were amplified using Axoclamp 2B and 900A amplifiers (Molecular Devices). We discarded all muscle fiber recordings in which the resting membrane potential was more depolarized than −55 mV, and all PD soma recordings in which the trough potentials were more depolarized than −50 mV.

For current clamp recordings, a single muscle fiber was impaled with two electrodes at <1 mm distance, one for injection and one for recording. In a subset of these experiments, we used pharmacological manipulation; 5 mm tetraethylammonium (TEA; Fluka) was used to partially block potentially present voltage-gated or Ca^2+^-gated K^+^ currents, and 100 nm tetrodotoxin (TTX; Sigma-Aldrich) to block potentially present voltage-gated Na^+^ currents. To partially block potential Ca^2+^ currents (and therefore indirectly Ca^2+^-gated K^+^ currents), 90% of the CaCl_2_ in the saline was replaced with MnCl_2_.

Electrical nerve stimulation was achieved by placing a stainless-steel wire inside the stimulation well, and another one into the bath right next to the well. The leads were connected to an isolated pulse stimulator (AM Systems, model 2100). Pulse durations were between 200 and 500 μs. To mimic realistic bursting input, the generic pattern we used consisted of 19 stimuli per burst, in a parabolic interval structure, at a burst frequency of 1 Hz and a duty cycle (*DC*) of 0.35 (Ballo and Bucher, 2009; Ballo et al., 2012). This pattern was modified as indicated in the results section.

All electrophysiological signals were acquired using a micro1401 digitizing board (Cambridge Electronic Design) and the accompanying Spike2 software (versions 7–9). Stimulation protocols were generated using either the time settings of the stimulator, or the sequencer interface of the digital-to-analog converter of the micro1401, connected to the trigger input of the stimulator.

### Data analysis

Primary data analysis to extract timing and waveform parameters was performed using Spike2 and programs written in its script language. Recording traces were low pass filtered using the smoothing or median filter functions in Spike2. Care was taken that filtering only affected fast noise components and did not alter the amplitude or time course of physiological signals.

Summation in compound synaptic responses to repetitive input was dealt with in two different ways. For amplitude measurements of test responses following conditioning pulses or trains (Fig. 4), we subtracted a voltage template obtained from isolated single responses or responses to trains, scaled to the amplitude of the conditioning responses in each trial. In all other analyses, we exclusively measured voltage integrals (area above baseline) instead of amplitudes, as voltage integrals are independent of summation. In some cases, these responses were normalized for comparison between different stimulus regimes.

Secondary analyses, statistical tests, and plots were generated in SigmaPlot (version 12.0, Systat Software). If normally distributed, values are reported as means ± SEM or SD, as indicated. If not normally distributed, values are reported as medians with 95% confidence interval (CI). Not normally distributed data are shown in box-and-whisker plots, where the whiskers represent the 90^th^ and 10^th^ percentiles. Regression analyses were performed from fits of various functions, as indicated. For normally distributed data, we used paired *t* tests, unpaired *t* tests, and one-way or two-way repeated measures (RM)-ANOVA with subsequent Holm–Sidak *post hoc* comparisons where needed. For data not normally distributed, we used Mann–Whitney rank-sum tests, Wilcoxon signed-rank tests, and Friedman RM-ANOVA on ranks with subsequent Tukey’s *post hoc* comparisons where needed. Significance was assumed at *p *<* *0.05 and is indicated as *p <* *0.05, **p <* *0.01, and ***p <* *0.001. Figures were produced in Canvas (version 11, ACD Systems). All statistical tests are summarized in Table 1.

**Table 1:** Statistical tests

Source	Data structure	Type of test	Power (α = 0.05) or percentile [25%, 75%]
Stimulus-response delay (Fig. 1*D*)	Normal	Two-tailed paired *t* test	0.647
Input resistance (Fig. 3*A*)	Normal	Two-tailed *t* test	0.120
Train-pulse undershoot (Fig. 4*C*)	Normal	Two-tailed paired *t* test	0.555
EJP halfwidth (Fig. 4*D*)	Normal	Two-tailed *t* test	1.000
Constant *DC*, *cpv2a* (Fig. 7*B*)	Non-normal	Friedman RM-ANOVA on ranks	*F*_burst_ = 0.20 Hz: [−31.613, −13.993] *F*_burst_ = 0.27 Hz: [−31.515, −10.355] *F*_burst_ = 0.36 Hz: [−24.600, −9.441] *F*_burst_ = 0.47 Hz: [−13.663, −2.967] *F*_burst_ = 0.63 Hz: [−2.388, 13.840] *F*_burst_ = 0.84 Hz: [14.686, 35.370] *F*_burst_ = 1.12 Hz: [21.986, 47.462] *F*_burst_ = 1.50 Hz: [−12.216, 27.990]
Constant duration, *cpv2a* (Fig. 7*B*)	Non-normal	Friedman RM-ANOVA on ranks	*F*_burst_ = 0.20 Hz: [−21.500, −8.653] *F*_burst_ = 0.27 Hz: [−20.058, −7.656] *F*_burst_ = 0.36 Hz: [−19.780, −7.665] *F*_burst_ = 0.47 Hz: [−14.000, −5.313] *F*_burst_ = 0.63 Hz: [−8.894, −0.563] *F*_burst_ = 0.84 Hz: [0.206, 12.912] *F*_burst_ = 1.12 Hz: [10.114, 29.104] *F*_burst_ = 1.50 Hz: [15.022, 50.229]
Constant cycle period, *cpv2a* (Fig. 7*B*)	Non-normal	Friedman RM-ANOVA on ranks	*F*_spk_ = 10.0 Hz: [−18.274, −3.291] *F*_spk_ = 13.4 Hz: [−14.532, −0.546] *F*_spk_ = 17.9 Hz: [−13.996, −2.860] *F*_spk_ = 23.9 Hz: [−6.387, 5.362] *F*_spk_ = 31.8 Hz: [0.366, 7.156] *F*_spk_ = 42.4 Hz: [3.614, 13.879] *F*_spk_ = 56.6 Hz: [5.703, 25.047] *F*_spk_ = 75.4 Hz: [−9.573, 9.961]
Constant *DC*, *cpv2b* (Fig. 7*C*)	Non-normal	Friedman RM-ANOVA on ranks	*F*_burst_ = 0.20 Hz: [−66.951, −49.180] *F*_burst_ = 0.27 Hz: [−45.325, −9.273] *F*_burst_ = 0.36 Hz: [−12.622, 29.986] *F*_burst_ = 0.47 Hz: [37.380, 59.705] *F*_burst_ = 0.63 Hz: [41.814, 89.525] *F*_burst_ = 0.84 Hz: [16.048, 47.712] *F*_burst_ = 1.12 Hz: [−45.898, 2.245] *F*_burst_ = 1.50 Hz: [−74.853, −61.184
Constant duration, *cpv2b* (Fig. 7*C*)	Non-normal	Friedman RM-ANOVA on ranks	*F*_burst_ = 0.20 Hz: [−11.148, 15.365] *F*_burst_ = 0.27 Hz: [−10.030, 19.313] *F*_burst_ = 0.36 Hz: [−16.128, 7.812] *F*_burst_ = 0.47 Hz: [−23.693, −8.204] *F*_burst_ = 0.63 Hz: [−17.809, −8.542] *F*_burst_ = 0.84 Hz: [−11.823, 2.310] *F*_burst_ = 1.12 Hz: [7.066, 29.300] *F*_burst_ = 1.50 Hz: [−7.319, 29.727]
Constant cycle period, *cpv2b* (Fig. 7*C*)	Non-normal	Friedman RM-ANOVA on ranks	*F*_spk_ = 10.0 Hz: [−66.870, −35.296] *F*_spk_ = 13.4 Hz: [−43.318, −6.218] *F*_spk_ = 17.9 Hz: [−16.198, 25.770] *F*_spk_ = 23.9 Hz: [10.557, 49.596] *F*_spk_ = 31.8 Hz: [33.029, 75.238] *F*_spk_ = 42.4 Hz: [5.421, 56.999] *F*_spk_ = 56.6 Hz: [−29.973, −1.063] *F*_spk_ = 75.4 Hz: [−56.215, −29.974]
19–17 pulses, *cpv2a* (Fig. 8B)	Normal	One-way RM-ANOVA	1.000
19–17 pulses, *cpv2b* (Fig. 8B)	Normal	One-way RM-ANOVA	1.000
Parabolic vs regularized, *cpv2a* (Fig. 9B)	Normal	Two-tailed paired *t* test	0.052
Parabolic vs regularized, *cpv2b* (Fig. 9B)	Non-normal	Wilcoxon signed-rank test	Regularized: [1.202, 2.424] Parabolic: [1.192, 2.607]
CV parabolic vs regularized, *cpv2a* (Fig. 9*C*)	Non-normal	Wilcoxon signed-rank test	Regularized: [0.037, 0.060] Parabolic: [0.038, 0.081]
CV parabolic vs regularized, *cpv2b* (Fig. 9*C*)	Normal	Two-tailed paired *t* test	0.838
Spike interval patterns, cycle-by cycle, *cpv2a* (Fig. 10*B*)	Non-normal	Friedman RM-ANOVA on ranks	control: [−0.230, 0.535] -Ih: [−3.980, −1.970] DA: [1.915, 3.540]
Spike interval patterns, cycle-by cycle, *cpv2b* (Fig. 10*B*)	Normal	One-way RM-ANOVA	1.0
Spike interval patterns, 5 cycles, *cpv2a* (Fig. 10*B*)	Non-normal	Friedman RM-ANOVA on ranks	control: [−0.142, 0.793] -Ih: [−3.967, −1.872] DA: [1.875, 3.443]
Spike interval patterns, 5 cycles, *cpv2b* (Fig. 10*B*)	Normal	One-way RM-ANOVA	1.0

## Results

### Muscle responses to spontaneous rhythmic input differed substantially between *cpv2a* and *cpv2b*

In an initial set of experiments, we kept most of the STNS intact and connected to the PD innervated muscles. We simultaneously recorded from a PD soma and a muscle fiber, either *cpv2a* (*n* = 7) or *cpv2b* (*n* = 6), during ongoing rhythmic pyloric activity (Fig. 1*C*,*D*). In *H. americanus*, pyloric rhythm frequencies range from 0.4 to 1 Hz (Bucher et al., 2005, 2006). PD neurons are parabolic bursters, producing ∼10–25 spikes per burst, yielding intra-burst mean spike frequencies of 10–35 Hz, and peak instantaneous spike frequencies of 50–100 Hz (Ballo and Bucher, 2009). Soma recordings show waveforms consisting of substantially attenuated spikes on top of only minimally attenuated slow wave depolarizations, as is typical for STG neurons (Mulloney and Selverston, 1972; Golowasch and Marder, 1992).

Stomach muscle fibers are multiterminally innervated along their length, which ensures spatial spread of synaptic potentials (excitatory junction potentials; EJPs) because they usually do not produce all-or-none overshooting action potentials. They generate slow and graded contractions in response to EJPs that summate during bursting input (Morris and Hooper, 1998), and have fairly homogeneous properties within a given muscle (Govind and Lingle, 1987). Both *cpv2a* and *cpv2b* showed summated depolarizations of a few millivolts, albeit with very different dynamics. In *cpv2a*, responses to bursting input comprised easily discernible individual EJPs (Fig. 1*D*). The responses were fast in the sense that they included a rapid depolarization at the onset. Fibers of stomach muscles innervated by more than one neuron are often polyneural, meaning that individual fibers receive input from more than one motor neuron (Govind and Lingle, 1987; Daur et al., 2012). Individual fibers of *cpv2a* appeared to receive input from both PD neurons. PD neurons are strongly electrically coupled in the STG, but while this causes highly coherent slow wave oscillations, spike initiation occurs independently in both neurons (unpublished results). Therefore, spikes are not synchronous and arrive at the muscle with some timing offset. This caused a higher number of distinguishable EJPs than could be accounted for by a single PD neuron’s spike pattern, and the varying timing offset led to varying patterns of summation (Fig. 1*D*, black arrow).

In *cpv2b*, initial depolarization was much slower than in *cpv2a*, and occurred with substantial delay from the onset of the PD burst. Peak depolarization was only reached toward the end of burst input, or even after. Individual EJP responses were not easily discernible, and often completely undetectable. Single burst responses could have a relatively smooth appearance in some phases (Fig. 1*D*, white arrow) and could show more distinct local peaks in others (Fig. 1*D*, gray arrow), possibly also because of changing offsets between the arrival times of spikes from both PD neurons.

In four experiments, we sequentially recorded from both *cpv2a* and *cpv2b* and could therefore directly compare the delay between PD spikes in the STG and the onset of individual muscle fiber responses (Fig. 1*D*, dashed lines and double-sided arrows). Despite recording sites at similar distance to the proximal muscle insertion sites (Fig. 1*B*), delays were significantly different (*cpv2a*: 48.8 ± 2.0 ms SEM; *cpv2b*: 75.2 ± 6.6 ms SEM; paired *t* test: *t*_(3)_ = −3.482, *p *=* *0.04). As conduction delay between STG and the proximal pdn is between 40 and 50 ms (Bucher et al., 2005), the delay to *cpv2a* activation is consistent with a direct path of the axon branches to the proximal part of the muscle fibers. However, the ∼25-ms longer delay for *cpv2b* may indicate a more circuitous path, possibly projecting to a more distal part of the fibers first and then returning to more proximal sites.

### Postsynaptic nonlinear muscle fiber membrane properties differed between *cpv2a* and *cpv2b*

For the remainder of the study, the muscles were disconnected from centrally generated input by cutting the nerves, and synaptic potentials were evoked by electrically stimulating the pdn. In all cases, stimulus voltage was set to be substantially above threshold to ensure that both PD axons were stimulated synchronously at all frequencies. This differs from the slight offsets in spike timing between the two PD axons during spontaneous pyloric activity (Fig. 1*D*). However, given the small amplitudes of total responses to even full bursting input, the difference this could make for summation and local voltage values is likely in the submillivolt range.

**Figure 2.**
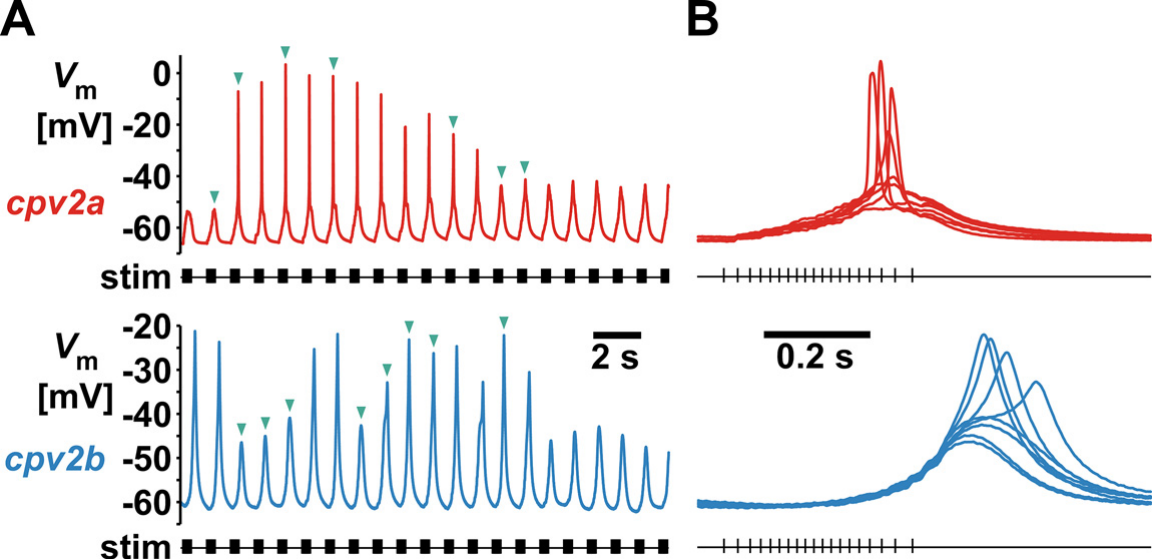
GAPs in *cpv2a* and *cpv2b*. ***A***, Episodes of GAPs in recordings from different experiments. Synaptic responses are from pdn stimulation with a regular parabolic burst pattern at *F*_burst_ = 1 Hz. ***B***, Overlay of all burst responses marked by teal arrowheads in ***A***, showing different depolarization slopes.

In some of the experiments, we observed graded action potentials (GAPs; Fig. 2*A*) when the pdn was stimulated with a burst pattern of parabolic interval structure (see Materials and Methods). GAPs are amplifying potentials of varying amplitudes that are common in some arthropod skeletal muscles (Araque and Buño, 1995; Araque et al., 1998; Weiss et al., 2001). They were straightforward to distinguish from regular EJPs not just by their larger amplitudes, but also by their steep voltage slopes to peak (Fig. 2*B*). GAPs were seen in only a few experiments in *cpv2a* (12/202 animals = 6%) but were more common in *cpv2b* (119/216 animals = 55%). In those experiments, they only occurred episodically and did not show a consistent dependence on presynaptic spike rates, as even during constant repetitive bursting input their occurrence waxed and waned (Fig. 2*A*). It was therefore straightforward to exclude them from analysis of synaptic responses shown in the remainder of this study. However, they were a clear indication that both muscles exhibited nonlinear voltage responses to synaptic currents.

**Figure 3.**
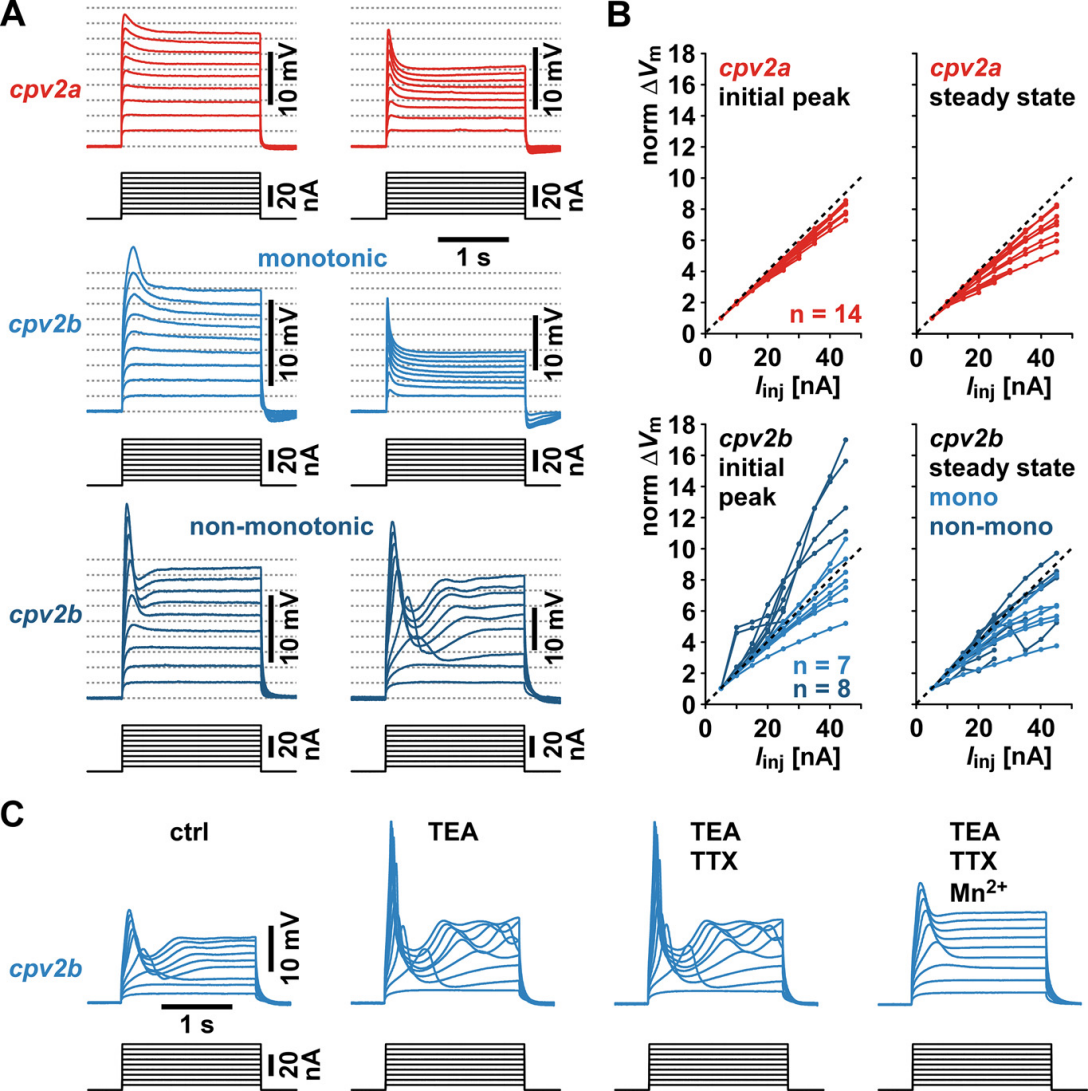
Step current injections into *cpv2a* and *cpv2b* fibers in two-electrode current clamp. ***A***, Example responses to 2-s steps of depolarizing current injections, in increments of 5 nA, from 5 to 45 nA. All responses are shown scaled to the same height of the smallest voltage response (5-nA current injection). Dashed lines indicate integer multiples of that first response. The two examples of *cpv2a* responses show different rates of monotonic decay from an initial peak in the voltage responses to larger current amplitudes. Two of the four *cpv2b* examples (lighter blue) show similar monotonic decay at different rates. The other two (darker blue) show amplified initial peak responses followed by non-monotonic trajectories. ***B***, Normalized V-I curves from individual experiments (*cpv2a*: *n* = 14; *cpv2b*: *n* = 15, 7 with monotonic and 8 with non-monotonic trajectories following the peak). In some experiments, a more limited range of current steps was used. Curves were obtained from the amplitudes of the initial voltage peaks and the mean voltage of the last ∼200 ms of each step (“steady state”). In each experiment, voltage amplitude was scaled to the smallest responses (5-nA current injection) and plotted as a function of current. Therefore, values below the identity line indicate that the voltage response was smaller than the corresponding integer multiple of the smallest response, and values above indicate that the response was larger. ***C***, Pharmacological manipulation of response properties in an example *cpv2* recording; 5 nm TEA and 100 nm TTX were added sequentially. In the last step, Ca^2+^ in the saline was reduced to 10% of the normal value, the rest replaced with Mn^2+^.

To gauge the degree of postsynaptic membrane nonlinearities, we performed dual impalements and current injections into single fibers. We did not precisely determine the fiber size distribution, but cursory estimates suggested that diameters exceeded 50 μm in both muscles. Correspondingly, fiber input resistances were very low. Voltage responses to 5-nA current injections yielded apparent input resistance values of 0.53 MΩ (±0.06 SEM, *n* = 14) in *cpv2a*, and 0.62 MΩ (±0.10 SEM, *n* = 15) in *cpv2b*. These values were not statistically different from each other (*t* test: *t*_(27)_ = −0.797, *p *=* *0.216). Because of the large currents needed to change the membrane potential, and the substantial space constants, we could not achieve voltage clamp of sufficient quality to measure voltage-gated currents reliably. However, step currents of increasing amplitudes revealed clear transients in the voltage responses. These nonlinearities were variable across recordings. Figure 3*A* shows different examples from each muscle. In *cpv2a*, initial voltage transients in response to larger current amplitudes always decayed monotonically to steady state, albeit at varying rates (Fig. 3*A*, upper traces). In *cpv2b*, 7 of 15 experiments showed transients that decayed monotonically, similar to *cpv2a* (Fig. 3*A*, middle traces). In the remaining 8 experiments, the initial transients were larger and decayed non-monotonically, either just producing a trough before reaching steady state (Fig. 3*A*, lower left), or eliciting moderate membrane oscillations (Fig. 3*A*, lower right).

Figure 3*A* also shows that voltage responses did not increase linearly with current. In all *cpv2a* recordings, both peak and steady-state responses to larger currents were smaller than the linear extrapolation of responses to the smallest current injection (Fig. 3*A*, dashed lines). In *cpv2b*, peak responses could be either smaller or larger than the linear extrapolation of responses to the smallest current injection, but steady-state responses were smaller in most cases. We plotted the data from all individual experiments as normalized V-I curves for peak and steady-state responses, in which the identity line indicates linear increase from the smallest response. For *cpv2a*, all V-I curves fall below the identity line, slightly at the initial peak and more substantially at steady state. This suggests that the responses were dominated by outward currents, and that at least parts of the outward currents were activated slowly. In *cpv2b*, V-I curves differed between experiments with monotonically or non-monotonically decaying transients. The former also fell mostly below the identity line, both at the peak and at steady state, whereas all the latter examples were above the identity line at peak. This suggests that in those experiments, the peaks were amplified by inward currents.

To gain further insight into potential ionic mechanisms, we performed pharmacological manipulations. In the example *cpv2b* recording shown in Figure 3*C*, application of a relatively low dose of TEA (5 mm), which blocks many voltage-gated and some Ca^2+^-gated K^+^ channels (Lang and Ritchie, 1990; Golowasch and Marder, 1992), amplified the initial transient response and the following membrane oscillations. Adding TTX did not change the responses, indicating that inward currents likely responsible for the enhanced peaks were not carried by TTX-sensitive voltage-gated Na^+^ channels. Replacing 90% of the Ca^2+^ with Mn^2+^ (in the continued presence of TEA and TTX) eliminated the amplification of the initial peaks and the membrane oscillations. Initial transients were still present and steady state responses were larger than in control saline. We only performed a qualitative assessment of the effects, for two reasons. First, the magnitude of the effects was variable across experiments and appeared to largely depend on how much nonlinearity was expressed in control saline. In recordings that displayed a monotonic decay of the initial transient (all *cpv2a* recordings and a subset of *cpv2b* recordings), TEA generally caused a more subtle increase in voltage amplitude and no obvious increases in non-linearities. Second, TEA amplification of peaks in some experiments was associated with fiber contractions resulting in movement artifacts that prevented assessment of the responses to the full range of current amplitudes. This was particularly apparent when TEA led to large repetitive GAP-like oscillations. However, qualitatively, responses were similar across experiments (*cpv2a*, *n* = 9; *cpv2b*, *n* = 6).

We interpret the findings described in Figure 3 as an indication that both muscles express multiple voltage-gated and possibly Ca^2+^-gated currents. In *cpv2a*, intrinsic properties predominantly dampen responses, while membrane properties in *cpv2b* fibers can either dampen or amplify responses. Therefore, all synaptic dynamics explored in the remainder of this study likely resulted both from short-term synaptic plasticity and substantial nonlinear muscle fiber membrane properties.

### Magnitude and frequency dependence of short-term facilitation differed between *cpv2a* and *cpv2b*

**Figure 4.**
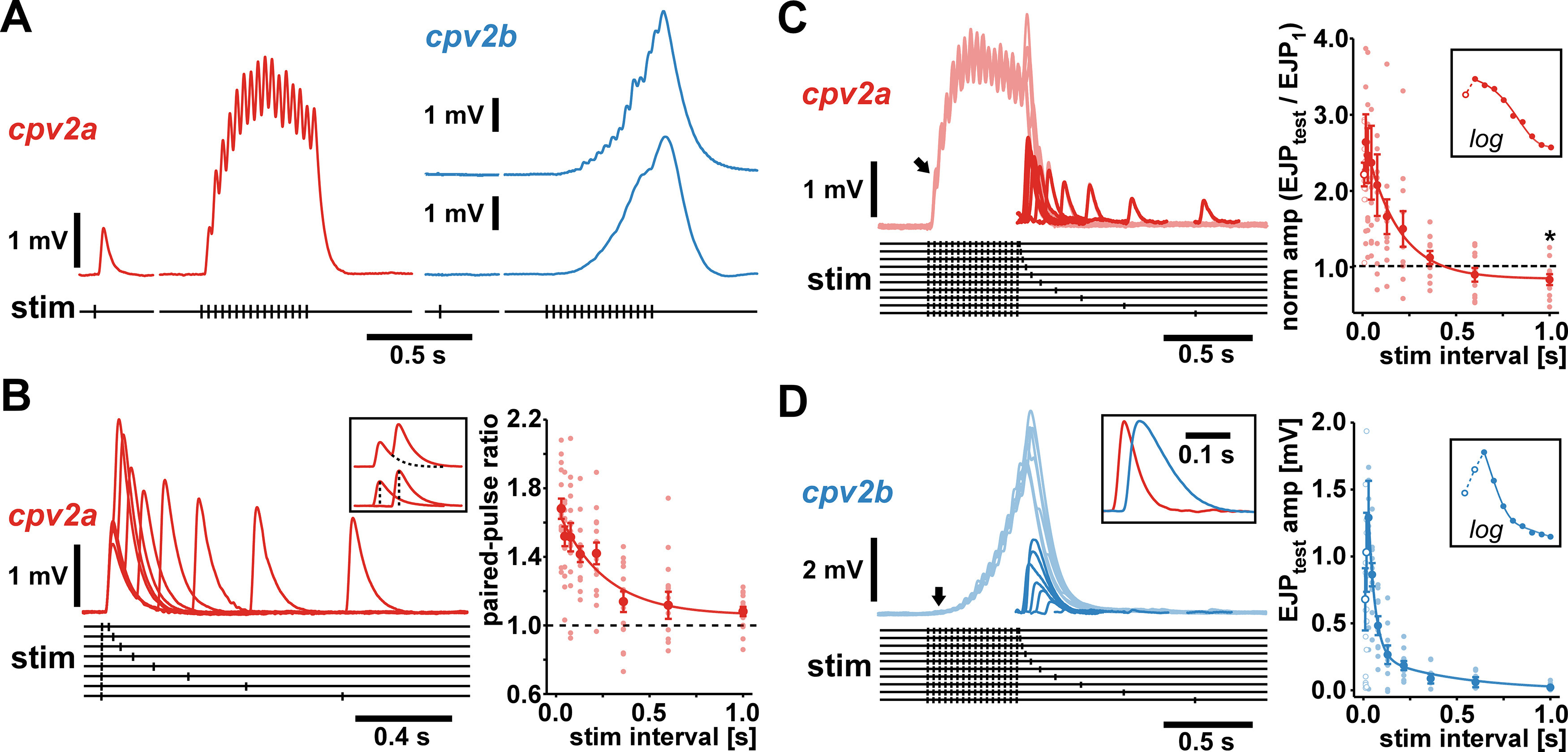
Short-term facilitation in response to stimulation of the pdn. ***A***, Responses to single and train stimulations (16 stimuli at 30 Hz) in a *cpv2a* fiber and two *cpv2b* fibers. ***B***, Paired-pulse facilitation in *cpv2a*. Multiple sweep traces show raw responses. For amplitude measurements, traces were corrected for summation (inset). The plot shows all individual data points across experiments (light red) as the amplitude ratio between response to test and conditioning pulse, as a function of stimulus interval (*n* = 15). Facilitation recovered as a single exponential decay (*R*_(120)_ = 0.648, *p *<* *0.0001, *τ* = 244 ms). Mean values ± SEM (dark red) are shown for clarity. ***C***, Responses to conditioning trains (16 stimuli at 30 Hz) and test pulses in *cpv2a*. Multiple sweeps of raw traces are shown in light red. Responses to test stimuli (dark red) were reconstructed by subtracting a scaled template of the response to the conditioning train. The plot shows all individual data points across experiments (light red) as the amplitude ratio of the responses to the test pulse and the first peak in the train response (arrow in the traces) as a function of stimulus interval (*n* = 11). Reponses at the shortest interval (10 ms, open circles) on average were smaller than at the second smallest interval (17 ms), as more easily discernible on a log scale (inset). At intervals >10 ms, facilitation recovered as a single exponential decay (*R*_(99)_ = 0.569, *p *<* *0.0001, *τ* = 176 ms). Mean values ± SEM (dark red) are shown for clarity. Decay undershot the initial response amplitude. At 1-s interval, the mean (not normalized) amplitudes of responses to the test pulse were significantly smaller than to the conditioning pulse (asterisk, paired *t* test, *t*_(10)_ = 2.324, *p *<* *0.05). ***D***, Responses to conditioning trains and test pulses in in *cpv2b*. Multiple sweeps of raw traces are shown in light blue, reconstructed responses to test stimuli in dark blue. The inset shows a single reconstructed response (blue) compared with the response of a *cpv2a* fiber (red, scaled to the same amplitude). As *cpv2b* usually did not exhibit discernible responses to the first conditioning stimulus (arrow), the plot shows all individual data points across experiments (light blue) as raw amplitudes (*n* = 10). Responses at the shortest intervals (10 and 17 ms, open circles) were smaller than at the next shortest (28 ms), as more easily discernible at on a log scale (inset). At intervals >17 ms, facilitation recovered as a double exponential (*R*_(80)_ = 0.789, *p *<* *0.0001, *τ*_1_ = 36 ms, *τ*_2_ = 440 ms). Mean values ± SEM (dark blue) are shown for clarity.

Crustacean stomach NMJs show substantial short-term synaptic plasticity, in most cases facilitation (Govind and Lingle, 1987; Sen et al., 1996; Jorge-Rivera et al., 1998; Stein et al., 2006; Daur et al., 2012; Blitz et al., 2017). To explore the dynamics of EJP responses, we used electrical stimulation of the pdn to activate PD axons in different patterns. Figure 4*A* shows responses to a single stimulus and a stimulus train. Two examples are shown for *cpv2b*, one with discernible individual responses, and one without. EJP responses to single stimuli in *cpv2a* had a median amplitude of 1.16 mV (0.50 CI, *n* = 15) but were absent or very small in *cpv2b* (*n* = 10).

Because *cpv2b* did not reliably respond to single stimuli but showed substantial responses to train input, it was evident that the NMJ undergoes substantial facilitation at this timescale. In contrast, *cpv2a* responded to single stimuli, but summation during train stimulation prohibited reliable assessment of short-term synaptic plasticity. We therefore performed a standard paired-pulse characterization on *cpv2a*, consisting of a single conditioning pulse and a test pulse delivered at varying intervals (Fig. 4*B*). We corrected for summation (Fig. 4*B*, inset; see Materials and Methods) and determined the paired-pulse ratio as a function of stimulus interval. The NMJ showed moderate facilitation, lasting for ∼1 s, and recovering as a single exponential decay function (*τ* = 244 ms).

To test whether more repetitive activity yields quantitatively different dynamics in *cpv2a* than with paired pulses, and to assess the time course of recovery in both *cpv2a* and *cpv2b*, we also used conditioning trains followed by a test pulse at varying intervals. We corrected for summation and determined the amplitudes of responses to the test pulses.

In *cpv2a* (Fig. 4*C*), we calculated the ratio between the test pulse and initial responses (Fig. 4*C*, arrow). Facilitation showed a more complex time course compared with the paired-pulse paradigm. Recovery was non-monotonic, as facilitation on average peaked only at the second smallest interval (28 ms). However, the remaining intervals showed recovery with a single exponential, albeit faster than with paired pulses (*τ* = 176 ms). In addition, recovery undershot the conditioning response (Fig. 4*C*, asterisk).

In *cpv2b* (Fig. 4*D*), we used raw EJP amplitudes in response to the test pulses because initial responses were mostly absent (Fig. 4*D*, arrow). The time course of facilitation was also non-monotonic. On average, facilitation peaked only at the third smallest interval (47 ms) and then recovered with a double exponential (*τ*_1_ = 36 ms, *τ*_2_ = 440 ms). We interpret the non-monotonic recovery from facilitation in both muscles, the undershoot in *cpv2a*, and the two recovery time constants present in *cpv2b* as an indication that more than one process contributed to the dynamics of the responses.

The reconstruction of unitary responses to test pulses in *cpv2b* also allowed comparison with *cpv2a* responses (Fig. 4*D*, inset). We confirmed the larger delay in *cpv2b* (as shown in Fig. 1*D*) and determined that the mean halfwidth was about twice as large (*cpv2a*: 52.6 ± 4.3 ms SEM, *n* = 14; *cpv2b*: 109.0 ± 4.2 ms SEM, *n* = 10; *t* test: *t*_(22)_ = −9.041, *p *<* *0.001). Therefore, more substantial summation likely explains the smoother appearance of *cpv2b* responses to bursts or trains.

**Figure 5.**
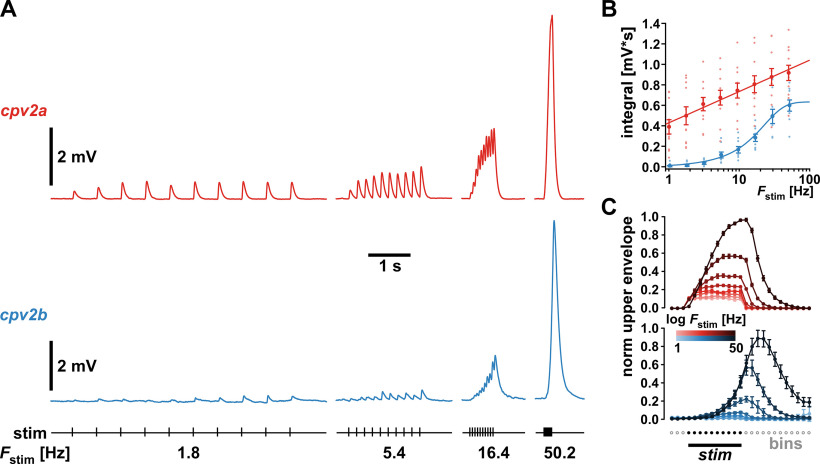
Facilitation across different stimulus frequencies. ***A***, Example traces of responses to trains of 10 stimuli delivered at different frequencies (*F*_stim_, 4 of 8 frequencies shown). ***B***, Responses quantified as the total voltage integral of each train response, showing all individual data points across experiments (lighter color) over the log of *F*_stim_ (*cpv2a*: *n* = 13; *cpv2b*: *n* = 10). Mean values ± SEM (darker colors) are shown for clarity. In both muscles, responses increased monotonically with *F*_stim_. In *cpv2a*, response increase with *F*_stim_ was fit with a logarithmic function (*R*_(88)_ = 0.575, *p *<* *0.0001). In *cpv2b*, response increase with *F*_stim_ was fit with a sigmoidal function (*R*_(72)_ = 0.892, *p *<* *0.0001). ***C***, Plots of the binned and normalized upper voltage envelopes from the same data shown in ***B***. For each train stimulus, voltage maxima were determined in bins set to the size of the stimulus interval, extending three bins before the first stimulus and 12 bins after the last. All responses within each experiment were normalized to the maximum depolarization at 50.2 Hz.

While the data presented in Figure 4 assessed facilitation as the dependence of unitary responses on prior activity, it did not address the dependence of compound responses on the rate of repetitive activity. Therefore, we also tested the responses to stimulus trains at varying frequencies (Fig. 5*A*). We stimulated the pdn with 10 pulses every 30 s, varying the stimulation frequency within trains (*F*_stim_) between 1 and 50.2 Hz. Figure 5*A* shows example traces from *cpv2a* and *cpv2b* recordings for some of the values of *F*_stim_. As a parameter that is independent of summation, we measured the voltage integral of each response to the whole train. Figure 5*B* shows that responses of the two muscles differed substantially in their frequency dependence, as *cpv2a* responses increased logarithmically with frequency, whereas *cpv2b* responses increased sigmoidally.

We also used these data to show that the time courses of depolarization in response to repetitive input were consistently different between *cpv2a* and *cpv2b*. Because individual peaks could not reliably be detected in *cpv2b* at all frequencies, and in *cpv2a* at higher frequencies, we measured the upper voltage envelopes by finding the voltage maxima in time bins that matched the stimulus interval at each frequency. Figure 5*C* shows the mean normalized upper voltage envelopes for all stimulus frequencies, plotted over the bin numbers. In *cpv2a*, envelopes showed an immediate and steep rise at stimulus onset and started to saturate before the end of the stimulus. In *cpv2b*, onset was delayed and gradual, and envelopes did not display any sign of saturation before the end of the stimulus and mostly peaked thereafter.

### Dynamics across bursts differed between *cpv2a* and *cpv2b*

**Figure 6.**
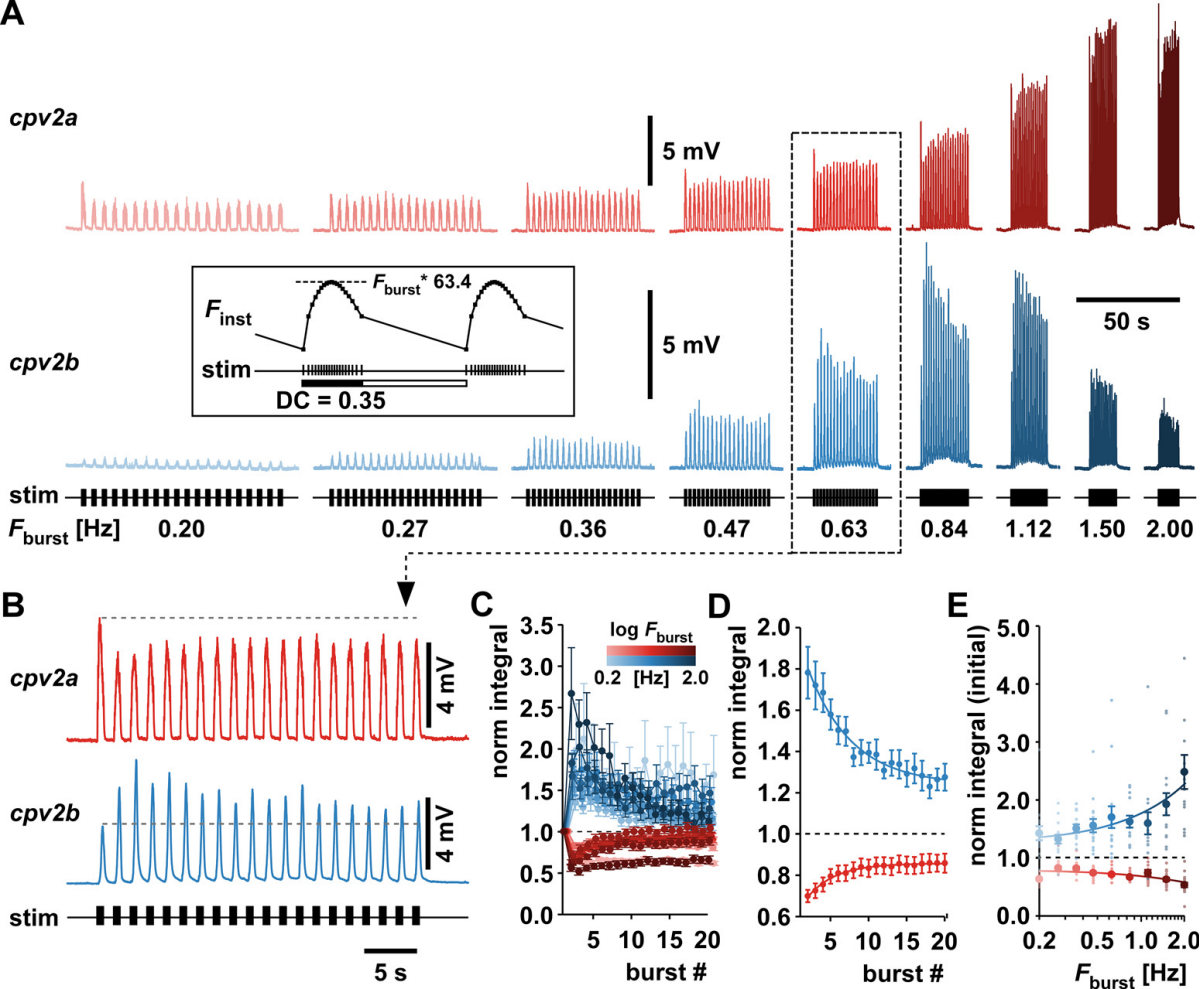
Dynamics across repeated bursting input. ***A***, Example responses to 20 consecutive bursts at different burst frequencies (*F*_burst_). The inset shows that single bursts consisted of 19 stimuli delivered in a parabolic interval structure (see Materials and Methods). Burst *DC* was kept constant at 0.35 across *F*_burst_ values. Maximum instantaneous frequency (*F*_inst_) scaled linearly as 63.4 * *F*_burst_. ***B***, Expanded view of the traces at *F*_burst_ = 0.63 Hz. Dashed lines indicate the amplitude of the first burst response. ***C***, Mean voltage integrals (normalized to the first burst response) as a function of burst index, for all values of *F*_burst_ (*cpv2a*: *n* = 17; *cpv2b*: *n* = 15). ***D***, Same normalized voltage integrals as in ***C***, as means of means across all burst frequencies, not individual data. The initial large changes in both muscles diminished to steady state as single exponential functions (*cpv2a*: *R*_(19)_ = 0.99, *p *<* *0.0001, rise constant = 3.7 bursts; *cpv2b*: *cpv2a*: *R*_(19)_ = 0.99, *p *<* *0.0001, decay constant = 6.7 bursts). ***E***, Magnitude of initial change as a function of *F*_burst_. Values are the average responses to the second and third bursts, normalized to the response to the first burst. Plots show all individual data points across experiments (small circles) over the log of *F*_stim_. Mean values ± SEM (large circles) are shown for clarity. Linear regression showed change with frequency at both NMJs (*cpv2a*: *R*_(153)_ = 0.43, *p *<* *0.0001; *cpv2b*: *R*_(135)_ = 0.44, *p *<* *0.0001).

Slower forms of synaptic plasticity like augmentation, post-tetanic potentiation, and long-lasting depression are only apparent with longer lasting and highly repetitive synaptic activation and can depend on a range of cellular phenomena (Regehr and Stevens, 2001; Zucker and Regehr, 2002). Pyloric neuron activity is highly repetitive, and the time constants of recovery from facilitation shown in Figure 4 suggested that dynamics could extend over multiple burst inputs, as has been shown for other stomach muscles (Stein et al., 2006; Blitz et al., 2017). We did not attempt to distinguish between cellular mechanisms that may be involved in dynamics apparent at different timescales, either presynaptically or postsynaptically. For simplicity, we therefore refer to any enhancement of synaptic responses as facilitation, and any decline as depression. We stimulated the pdn with a realistic parabolic burst pattern and recorded the responses to episodes of 20 consecutive bursts (Fig. 6*A*). We used the burst pattern described in Materials and Methods and varied the burst frequency (*F*_burst_) across episodes from 0.2 to 2 Hz. We did so while keeping the number of pulses per burst and the *DC* constant and scaling the inter-pulse intervals proportionally to the burst period (Fig. 6*A*, inset). In the example shown, response amplitudes increased with *F*_burst_ to some point but decreased again at higher values. This indicates bandpass filtering properties with lower best frequencies in *cpv2b* than in *cpv2a*. Amplitudes within each episode also changed. Therefore, differences in responses across different values of *F*_burst_ were clearly not simply caused by differences in summation. Summation across bursts even at higher values of *F*_burst_ was generally small, as seen in the only slight baseline depolarization during those episodes.

First, we assessed the dynamics across bursts within each episode. The expanded traces of the example recording at *F*_burst_ = 0.63 Hz (Fig. 6*B*) show that responses in *cpv2a* expressed initial depression across bursts, followed by partial recovery. In contrast, *cpv2b* expressed initial facilitation, followed by partial recovery. We measured the voltage integral of each burst response and normalized to the first response. Figure 6C shows the mean trajectory of normalized integrals over successive bursts for all values of *F*_burst_. Recovery was relatively consistent across values of *F*_burst_, apparently depending more on number of bursts than on time or frequency. We therefore pooled the data from all values of *F*_burst_ and fit the means with single exponentials (Fig. 6*D*). Responses in *cpv2b* took more bursts to recover (decay constant = 6.7 bursts) than responses in *cpv2a* (rise constant = 3.7 bursts). Although recovery appeared to be mostly dependent on burst number, the initial magnitude of depression or facilitation across burst responses was dependent on *F*_burst_. Figure 6*E* shows the ratios of the average response to the second and third bursts over the first burst. Both depression in *cpv2a* and facilitation in *cpv2b* increased significantly with *F*_burst_ (linear regression; *cpv2a*: *R*^2^ = 0.18, *p* < 0.0001; *cpv2b*: *R*^2^ = 0.19, *p* < 0.0001).

**Figure 7.**
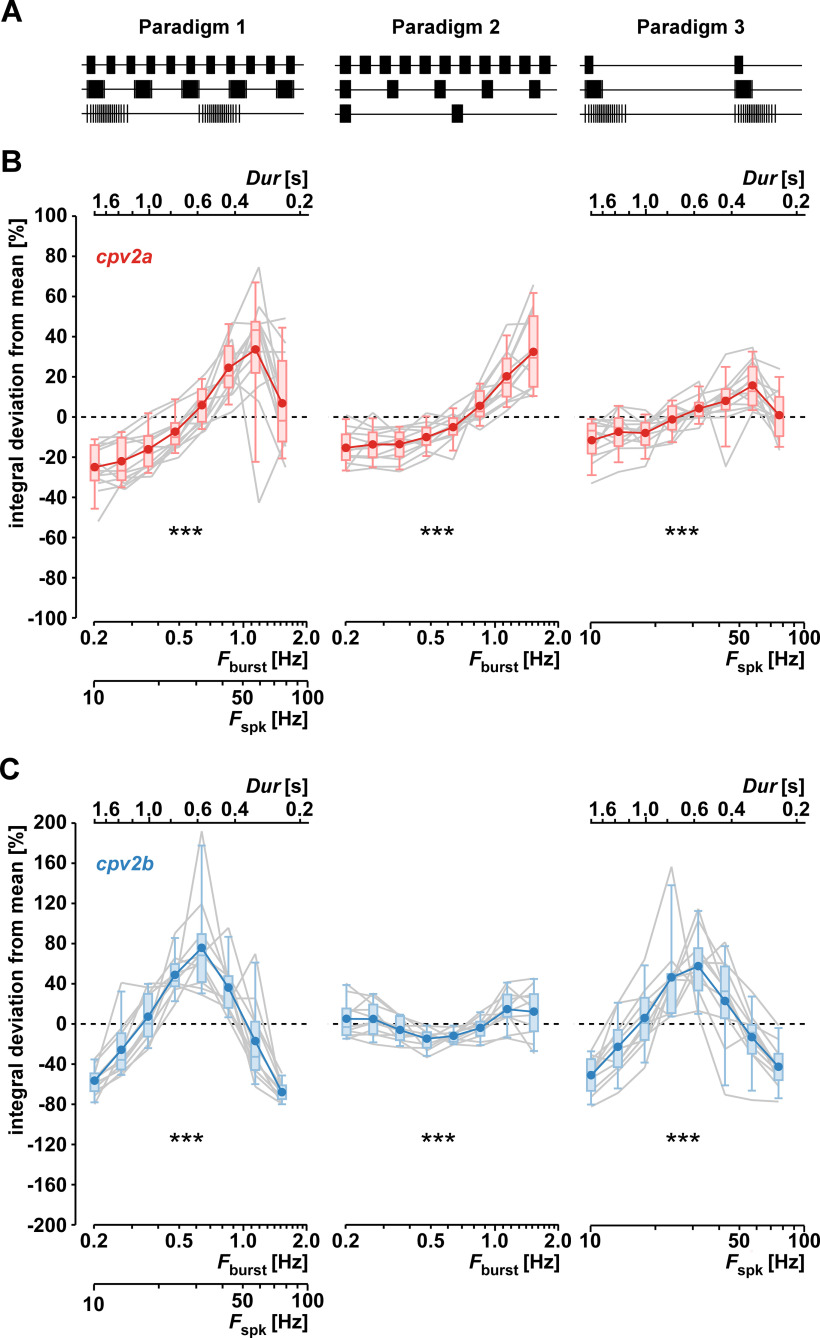
Sensitivity of responses to different burst parameters. ***A***, Three different stimulus paradigms used to distinguish between sensitivity to burst frequency (*F*_burst_) versus burst duration (*Dur*) and mean spike frequency within bursts (*F*_spk_). The number of spikes per burst was constant (19) in all cases. In paradigm 1, *DC* was constant while *F*_burst_ was varied between 0.2 and 1.5 Hz. Consequently, *F*_spk_ changed between 10.1 and 75.4 Hz, and *Dur* changed between 1.79 and 0.24 s. In paradigm 2, *F*_spk_ and *Dur* were constant at 55.6 Hz and 0.32 s, respectively, while *F*_burst_ was varied as in paradigm 1. In paradigm 3, *F*_burst_ was constant at 0.2 Hz, while *F*_spk_ and Dur changed as in paradigm 1. ***B***, ***C***, Steady-stated responses to the different stimulus paradigms (*cpv2a*: *n* = 13; *cpv2b*: *n* = 11). In each experiment, values were divided by the average response over all frequencies, and the results plotted as percent deviation from the overall mean. Asterisks indicate results from Friedman ANOVAs on ranks for each paradigm. Integrals changed significantly with *F*_burst_ and *F*_spk_ (*cpv2a*: χ^2^_(7)_ = 62.08, *p *<* *0.001; *cpv2b*: χ^2^_(7)_ = 60.87, *p *<* *0.001), *F*_spk_ (*cpv2a*: χ^2^_(7)_ = 72.43, *p *<* *0.001; *cpv2b*: χ^2^_(7)_ = 27.79, *p *<* *0.001) and *Dur* (*cpv2a*: χ^2^_(7)_ = 54.95, *p *<* *0.001; *cpv2b*: χ^2^_(7)_ = 55.24, *p *<* *0.001).

Next, we assessed the steady-state responses across frequencies from the average responses of the last five bursts (Fig. 7). As depicted in Figure 6*A*, inset, we had kept *DC* and number of spikes constant while changing *F*_burst_. Consequently, burst duration (*Dur*) changed inversely to *F*_burst_, while the mean spike frequency (*F*_spk_) changed proportionally. Any dynamics could therefore have resulted from the changes in *F*_burst_, *F*_spk_, or *Dur*. This paradigm was chosen because it reflects the PD neuron behavior across varying cycle frequencies (Bucher et al., 2005), and we refer to it from here on as paradigm 1. To distinguish between sensitivity of responses to *F*_burst_ versus *F*_spk_ or *Dur*, we applied two additional stimulus paradigms, which are shown alongside paradigm 1 in Figure 7*A*. Paradigm 2 was designed to keep *F*_spk_ and *Dur* constant while changing *F*_burst_. We fixed *Dur* at 0.32 s and *F*_spk_ at 55.6 Hz, corresponding to the value at *F*_burst_ = 1.1 Hz in paradigm 1. Paradigm 3 was designed to keep *F*_burst_ constant while changing *F*_spk_ and *Dur*. We fixed *F*_burst_ at 0.2 Hz but changed *F*_spk_ and *Dur* over the same range as in paradigm 1. We restricted the analysis to the *F*_burst_ range of 0.2−1.5 Hz, because in some experiments, *F*_burst_ = 2.0 Hz produced summation across bursts.

Figure 7*B* shows the change of mean voltage integrals in *cpv2a* under each stimulus paradigm. Under paradigm 1, voltage integrals increased with *F*_burst_ and *F*_spk_ over most of the range but decreased again at the highest frequency. This bandpass filtering property was absent under paradigm 2, but otherwise the magnitude of changes was similar. Under paradigm 3, the sensitivity to *F*_spk_ and *Dur* appeared less pronounced, but there was a decrease of integrals at the highest frequency similar to the responses to paradigm 1. We therefore conclude that the change in responses seen when burst parameters were changed in a realistic manner (paradigm 1) was because of changes in *F*_burst_ and either *F*_spk_ or *Dur*.

Figure 7*C* shows the change of mean voltage integrals in *cpv2b* under each stimulus paradigm. Under paradigm 1, voltage integrals showed pronounced bandpass filtering, with the largest responses at *F*_burst_ = 0.6 Hz and *F*_spk_ = 31.8 Hz. The magnitude of changes was substantially smaller under paradigm 2, and responses were smallest in the midrange of *F*_burst_ values. Under paradigm 3, changes were not as pronounced but qualitatively similar to the ones seen under paradigm 1, showing the same bandpass filtering. We therefore conclude that the change in responses seen when burst parameters were changed in a realistic manner (paradigm 1) was dominated by the changes in *F*_spk_ or *Dur*, and relatively insensitive to changes in *F*_burst_.

**Figure 8.**
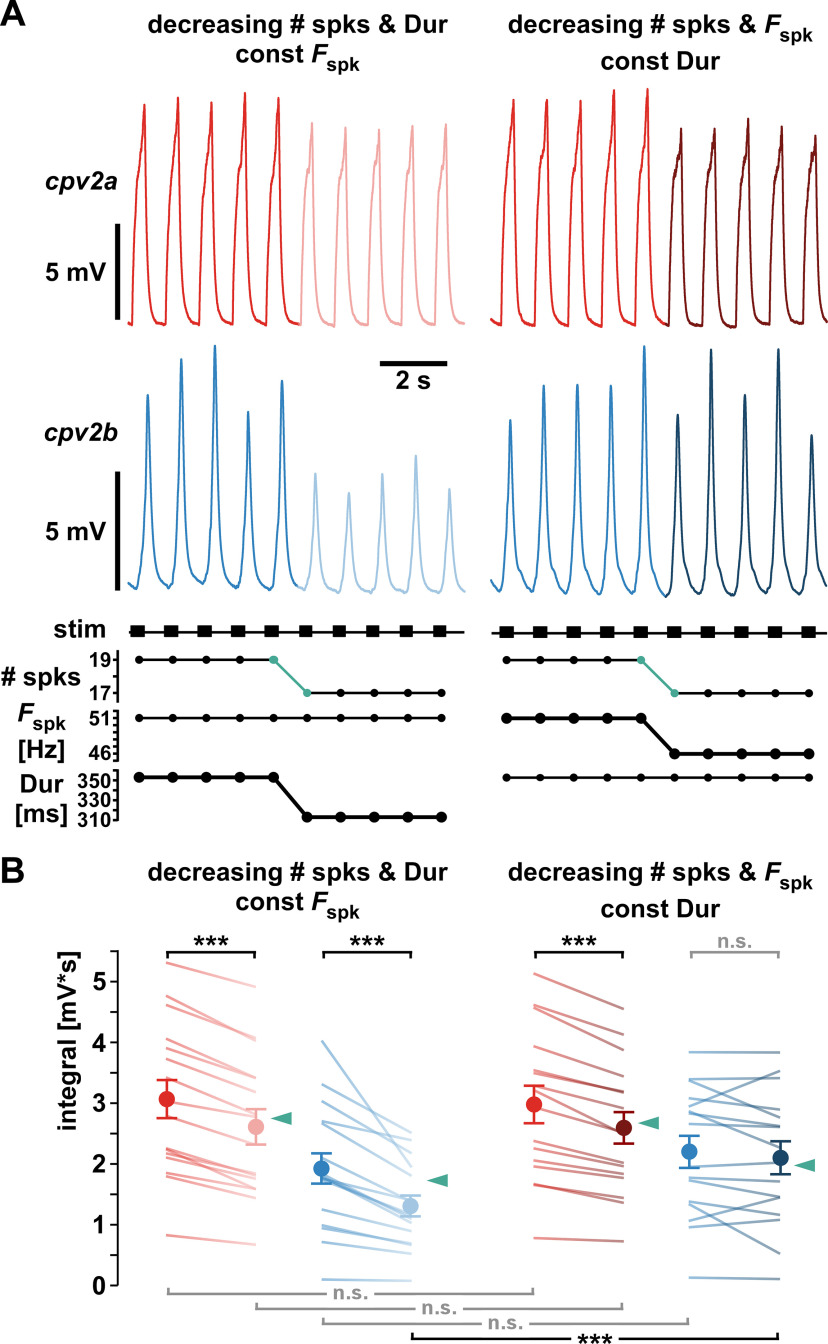
The effects of changing the number of pulses per burst. ***A***, Regularized burst patterns were changed from 19 pulses/burst (middle red or middle blue) to 17 pulses/burst (teal), first by decreasing *Dur* to keep *F*_spk_ constant (light red or light blue), and then by decreasing *F*_spk_ to keep *Dur* constant (dark red or dark blue). ***B***, Mean voltage integrals of the five responses before and five responses after the pattern changes. Teal arrowheads indicate the calculated mean linear decrease to 17/19 of the responses to 19 pulses/burst. In *cpv2a* (*n* = 16), responses differed between 19 and 17 pulses (one-way RM-ANOVA, *F*_(3,63)_ = 16.941, *p *<* *0.001). Responses decreased both when *Dur* and when *F*_spk_ changed, and the magnitude of decrease did not differ (Holm–Sidak *post hoc* comparisons). In *cpv2b* (*n* = 16), responses differed across stimulus regimes (one-way RM-ANOVA, *F*_(3,63)_ = 19.504, *p *<* *0.001), but only decreased significantly when *Dur* was decreased (Holm–Sidak *post hoc* comparisons).

The three paradigms used to change burst parameters did not allow us to distinguish between effects of *F*_spk_ and *Dur*. We therefore tested those effects in a different set of experiments. For simplicity, we used bursts of 19 pulses with constant (regularized) instantaneous frequency (*F*_inst_) at *F*_burst_ = 1 Hz and *Dur *=* *0.35 s. We stimulated for 20 s to reach steady state and then changed the pattern by reducing the number of pulses to 17. This drop in number of pulses was achieved in two ways, either by keeping *F*_spk_ constant and therefore decreasing *Dur* from 0.35 s to 0.31 s, or by keeping *Dur* constant and therefore decreasing *F*_spk_ from 51 to 46 Hz (Fig. 8*A*). We measured the mean voltage integral of the responses to the last five bursts before the change and the first five after and compared the results across conditions (Fig. 8*B*). In *cpv2a*, the decrease in number of pulses was accompanied by a significant decrease in voltage integral in both cases, and the effects of *Dur* and *F*_spk_ were statistically indistinguishable. In both cases, the decrease was close to the calculated linear decrease with pulse number (17/19 of the original response; Fig. 8*B*, teal arrowheads). In *cpv2b*, voltage integrals significantly decreased when *Dur* was decreased, but not when *F*_spk_ was decreased. The decrease in integrals with *Dur* was larger than the calculated linear decrease with pulse number (teal arrowheads). We conclude that responses in *cpv2a* depended on number of pulses in a burst, while responses in *cpv2b* only depended on *Dur*.

### Sensitivity to spike interval patterns within bursts differed between *cpv2a* and *cpv2b*

**Figure 9.**
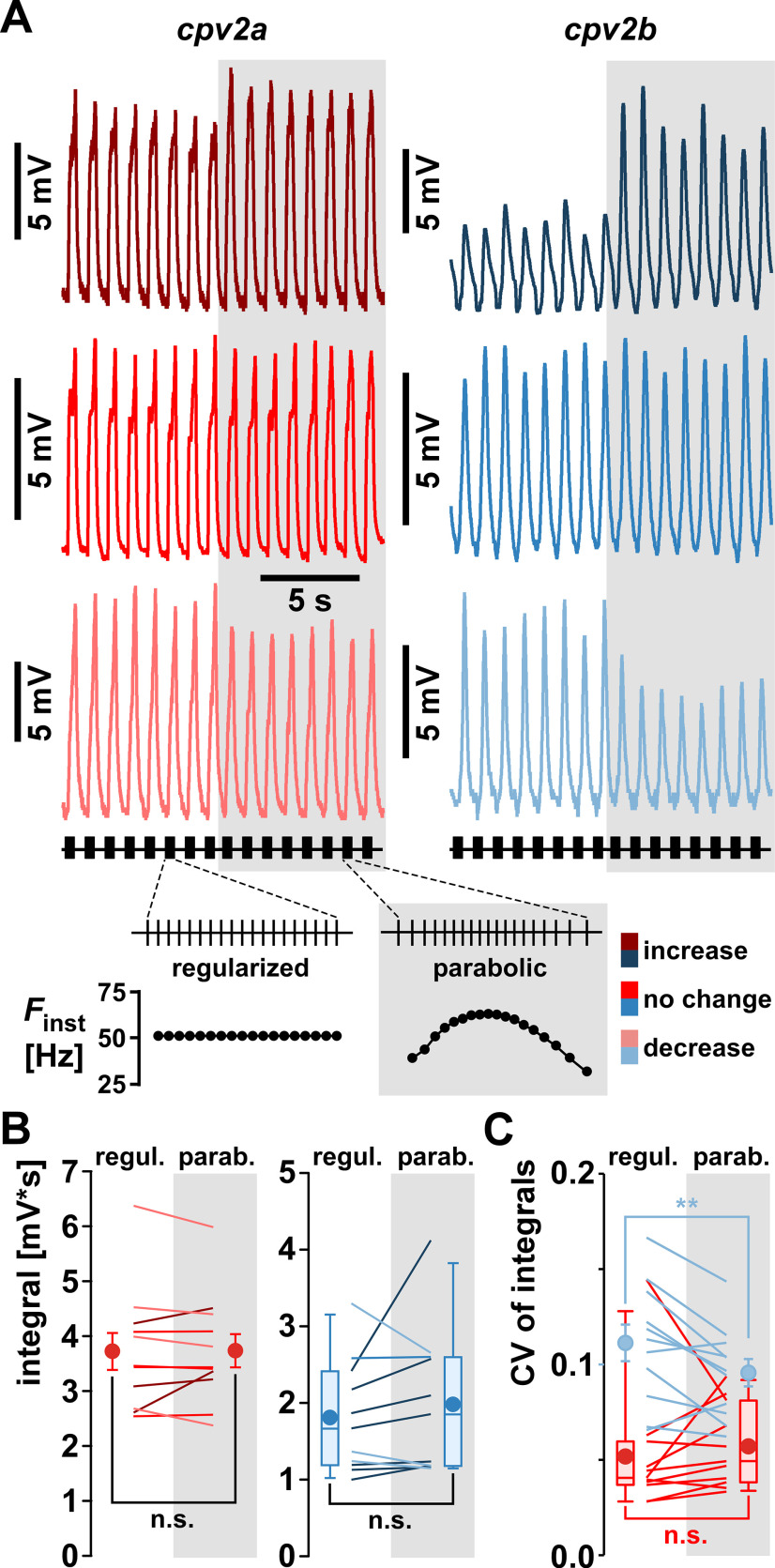
The effect of regularized versus parabolic intraburst stimulus patterns. ***A***, Examples of *cpv2a* and *cpv2b* recordings showing increasing (upper traces), stable (middle traces), and decreasing (lower traces) responses during a switch from regularized (constant instantaneous frequency, *F*_inst_) to parabolic patterns. ***B***, Mean integrals across experiments (*cpv2a*: *n* = 11; *cpv2b*: *n* = 11) did not significantly change between regularized and parabolic stimulation in either muscle (*cpv2a*: paired *t* test, *t*_(10)_ = 0.90, *p *=* *0.900; *cpv2b*: Wilcoxon signed-rank test, *Z*_(11)_ = 1.245, *p *=* *0.240). However, *t* tests or Mann–Whitney rank-sum tests of mean integrals from 50 to 60 cycles for each stimulus pattern in individual experiments (details not reported here) showed either increases (dark color lines), no change (middle color lines), or decreases (light color lines). ***C***, Cycle-to-cycle variability (CV) of response integrals. Lines show changes in individual experiments, and open circles (±SEM) mean CVs across experiments. Means were not significantly different in *cpv2a* (Wilcoxon signed-rank test, *Z*_(11)_= 1.600, *p *=* *0.123) but were significantly smaller for parabolic stimulation in *cpv2b* (paired *t* test, *t*_(10)_ = 3.273, *p *<* *0.01).

The results presented in Figures 7, 8 addressed the sensitivity of both NMJs to different burst parameters, but not to the precise temporal pattern within each burst. Pyloric neurons produce cell type-specific characteristic intraburst spike patterns that are malleable to neuromodulation (Szücs et al., 2003, 2005; Ballo and Bucher, 2009; Ballo et al., 2012; Zhang et al., 2017). To which degree muscle responses may be sensitive to those patterns, and changes thereof, is not known. During ongoing pyloric activity, the PD neuron shows a parabolic burst pattern (Szücs et al., 2003; Ballo and Bucher, 2009) We therefore tested whether the parabolic trajectory of *F*_inst_ within each burst was important in determining synaptic responses. We stimulated the pdn with a bursting pattern (19 pulses, *Dur *=* *0.35 s, *F*_burst_ = 1 Hz), alternating 1 min episodes of a regularized (constant *F*_inst_) intraburst pattern with the parabolic pattern. Figure 9*A* shows three examples for each muscle, indicating that the responses to changing the pattern were inconsistent across experiments. In some cases, responses increased when the pattern switched from regularized to parabolic (upper traces), in some cases there was no clear effect (middle traces), and in other cases responses decreased (lower traces). Figure 9*B* shows that in *cpv2a*, although there was no significant change in mean responses across experiments, 3 of 11 experiments showed an increase within the experiment, 4 showed a decrease, and another 4 showed no change. In *cpv2b*, mean responses across experiments also did not show a significant change, but 7 of 11 experiments showed an increase within the experiment, 3 showed a decrease, and one showed no change. We also wanted to test whether cycle-to-cycle variability was dependent on the pattern. To this end, we determined the coefficient of variation (CV) across cycles for each pattern in each individual experiment. Figure 9*C* shows that in *cpv2a*, mean cycle-to-cycle variability did not change, but in *cpv2b*, variability was smaller with parabolic stimulation.

**Figure 10.**
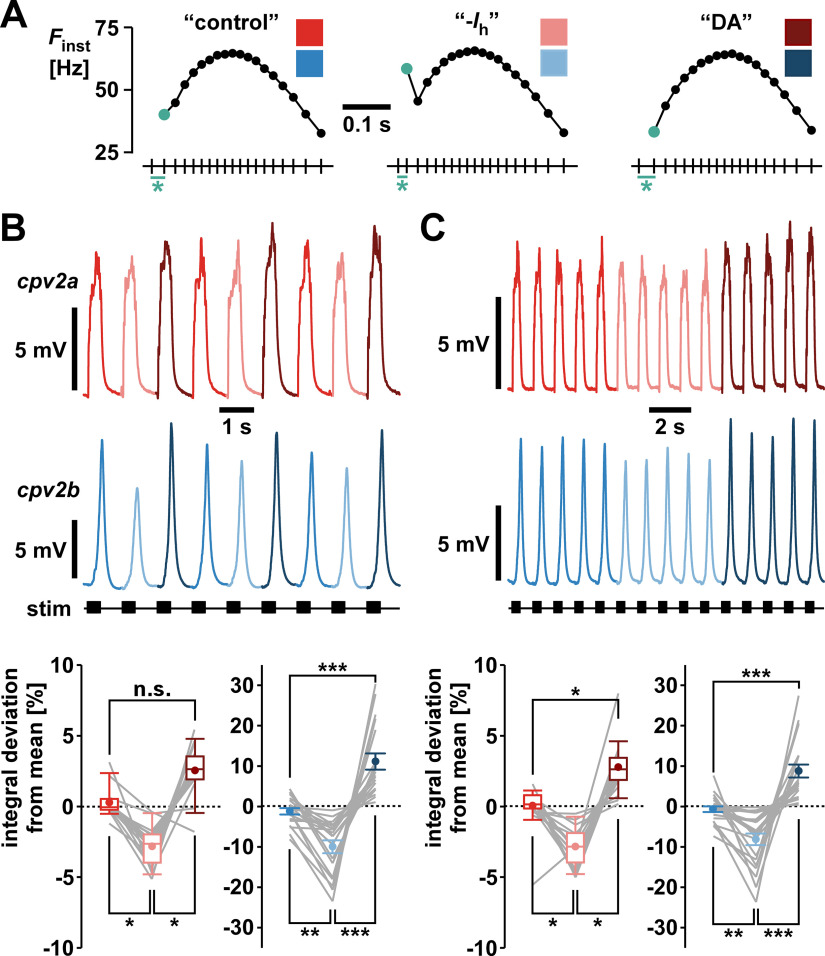
The effect of intraburst stimulus patterns mimicking pattern changes because of spike propagation. ***A***, Stimulus patterns corresponding to how the parabolic pattern used in the prior experiments changes during propagation from the STG to the muscle in control saline (control), when the hyperpolarization-activated inward current *I*_h_ is blocked (-*I*_h_), and when *I*_h_ is increased by DA. The most substantial difference between pattern at the beginning of the bursts is highlighted in teal. ***B***, Responses to cycle-by-cycle change of intraburst patterns, color coded as indicated in ***A***. Plots show mean normalized voltage integrals (*cpv2a*: *n* = 17; *cpv2b*: *n* = 19). In each experiment, at least 20 responses to each stimulus pattern were averaged. Each average value was then divided by the average across all stimulus patterns, and the results plotted as percent deviation from the overall mean. In *cpv2a*, the overall effect of stimulation pattern was significant (Friedman RM-ANOVA on ranks, χ^2^_(2)_ = 25.53, *p *<* *0.001), but control and DA were not significantly different (Tukey’s test). In *cpv2b*, the overall effect of stimulation pattern was also significant (one-way RM-ANOVA, *F*_(2,56)_ = 31.198, *p *<* *0.001), and responses differed between all patterns (Holm–Sidak *post hoc* comparisons). ***C***, Responses to episodes of five consecutive bursts for each pattern. Plots show normalized values from the averages of the last three of the five cycles in at least 10 episodes. Normalization was done and plots generated as described in ***B*** (*cpv2a*: *n* = 18; *cpv2b*: *n* = 22). In *cpv2a*, the overall effect of stimulation pattern was significant (Friedman RM-ANOVA on ranks, χ^2^_(2)_ = 32.11, *p *<* *0.001), and responses differed between all patterns (Tukey’s test). In *cpv2b*, the overall effect of stimulation pattern was also significant (one-way RM-ANOVA, *F*_(2,65)_ = 28.022, *p *<* *0.001), and responses differed between all patterns (Holm–Sidak *post hoc* comparisons).

In the PD neurons, activity-dependent changes in axonal excitability and conduction velocity affect the interval structure within bursts during propagation from the STG to the muscle (Ballo and Bucher, 2009). These changes are dependent on axonal hyperpolarization-activated inward current (*I*_h_), which is increased in the presence of dopamine (DA; Ballo et al., 2010). Blocking *I*_h_ exacerbates pattern changes because conduction velocity becomes more variable within each burst, while DA-mediated increase in *I*_h_ reduces pattern changes because conduction velocity becomes more consistent (Ballo et al., 2012; Zhang et al., 2017). We wanted to know whether such changes in interval structure affected muscle responses, particularly because they are more subtle than the difference between parabolic and regularized patterns. Figure 10*A* shows stimulus patterns that mimicked the burst patterns as they would arrive at the muscle in control saline (“control”), when *I*_h_ is blocked (“-*I*_h_”), and when *I*_h_ is increased by DA (“DA”; Ballo et al., 2012). The most prominent differences between the patterns are in the *F*_inst_ value at the beginning of the bursts, corresponding to the first interval (Fig. 10*A*, teal). In the -*I*_h_ pattern, the first interval is decreased, while in the DA pattern it is increased. First, we alternated the three stimulation patterns cycle by cycle (Fig. 10*B*). Both muscles were sensitive to the pattern changes, with generally smallest responses to the -*I*_h_ patterns, and largest responses to the DA pattern. However, in *cpv2a* only the responses to the -*I*_h_ pattern were significantly different from the others, and the difference was in the range of <5%. In contrast, responses to all patterns differed significantly from each other in *cpv2b*, and the differences could exceed 20%. To ensure that these differences were not just caused by transient changes because of the switching of patterns, we used two additional stimulus regimes. First, we still alternated the patterns cycle by cycle, but changed the sequence, which yielded similar results (data not shown). Second, we switched patterns only every five bursts and quantified responses from the average voltage integrals of the last three in each episode (Fig. 10*C*). Again, both muscles were sensitive to the differences in patterns, showing the smallest responses to the -*I*_h_ pattern, and the largest responses to the DA pattern. In this case, the differences were significant in all cases, and similar in magnitude to the cycle-by-cycle results. We conclude that the changes in *F*_inst_ within each burst that arise from activity-dependent changes in axonal spike propagation and are sensitive to axonal DA modulation are substantial enough to affect the magnitude of postsynaptic responses, particularly in *cpv2b*.

## Discussion

### Synapse-specific plasticity

The term short-term synaptic plasticity describes the activity dependence of synaptic currents. However, synaptic dynamics encompass changes in voltage responses to repetitive input that in addition are shaped by summation and by postsynaptic membrane nonlinearities arising from voltage-gated currents. We controlled for summation by measuring voltage integrals instead of just amplitudes but did not separate the effects of synaptic plasticity and membrane nonlinearities, as we measured synaptic responses in current clamp. Apart from the technical challenges of voltage-clamp recordings in muscle fibers with multiterminal innervation and large space constants, we did so because we wanted to characterize the actual response dynamics, not just the short-term plasticity of synaptic currents. That said, intrinsic membrane nonlinearities most likely serve to either enhance or dampen synaptic depolarization (Atwood, 1963; Araque and Buño, 1995; Araque et al., 1998), but are unlikely to account for the qualitative presence of facilitation and depression shown here. Instead, activity-dependent changes in synaptic responses are likely dominated by short-term presynaptic plasticity.

Most synapses show some form of short-term plasticity (Regehr and Stevens, 2001; Zucker and Regehr, 2002; Fioravante and Regehr, 2011; Regehr, 2012), and the resulting history and frequency dependence of synaptic transmission is an important part of neural coding in a variety of information processing contexts (Fortune and Rose, 2001; Abbott and Regehr, 2004; Klug et al., 2012; Anwar et al., 2017). Short-term synaptic plasticity comes in many different forms and is synapse type-specific, as it can qualitatively and quantitatively differ not just between synapses formed by different presynaptic neurons onto the same cell, but also between synapses formed by the same neuron onto different postsynaptic cells (Atwood and Karunanithi, 2002). Because most forms of short-term synaptic plasticity are predominantly presynaptic, the latter case requires differential regulation of presynaptic release properties by retrograde signaling from different postsynaptic cells (Davis and Murphey, 1993; Sylwestrak and Ghosh, 2012; Davis and Müller, 2015). In hippocampus and neocortex, the functional meaning of such synapse type-specific plasticity is not well understood but may play important roles in information processing and local circuit dynamics (Markram et al., 1998; Blackman et al., 2013; Larsen and Sjöström, 2015). In the brain stem, auditory nerve fibers that bifurcate to innervate different divisions of cochlear nuclei exhibit different short-term plasticity at their output synapses, which is thought to contribute to separate readout of intensity and timing cues of auditory signals encoded in the same presynaptic spike patterns (MacLeod, 2011). In crustacean skeletal muscles, different forms of short-term plasticity at NMJs formed by the same motor axon are correlated with the contraction properties of the postsynaptic fibers (Bittner, 1968; Atwood and Karunanithi, 2002).

In the crustacean stomach, synapse-specific dynamics has been shown before in muscles innervated by the same motor neuron. In one case, one NMJ showed facilitation and the other depression (Katz et al., 1993). In other cases, NMJs all facilitated, but magnitude and frequency dependence varied (Govind et al., 1975; Blitz et al., 2017). Such differences have not been systematically explored in the context of sensitivity to different attributes of neuron and circuit activity in the stomatogastric system. In the STG, neuromodulators sculpt versions of rhythmic circuit activity that differ in cycle frequency, relative timing, burst durations, and spike frequencies within bursts (Nusbaum et al., 2001; Dickinson, 2006; Marder and Bucher, 2007; Stein, 2009; Harris-Warrick, 2011; Nusbaum and Blitz, 2012). In addition, modulators control more subtle activity attributes like cycle-to-cycle regularity (Zhao et al., 2011), and spike interval structure within bursts (Szücs et al., 2003, 2005; Ballo and Bucher, 2009; Ballo et al., 2012). The differences in synaptic dynamics between *cpv2a* and *cpv2b* described here may be most relevant in the context of such changes in input patterns, through neuromodulation or other perturbations.

### Dynamics across spikes versus dynamics across bursts

Both *cpv2a* and *cpv2b* showed response dynamics dominated by facilitation, as is typical for stomach muscles (Govind and Lingle, 1987; Jorge-Rivera et al., 1998; Stein et al., 2006; Blitz et al., 2017). Apart from occasional GAPs, unitary EJPs waveforms, temporal summation, and facilitation in *cpv2a* were similar to synaptic responses in other pyloric muscles (Govind et al., 1975; Govind and Lingle, 1987; Pulver et al., 2005; Daur et al., 2012). In contrast, the near-absence of responses to single presynaptic stimuli and the delayed onset of burst responses in *cpv2b* was unusual for stomach muscles but reminiscent of facilitating responses in tonic fibers of crayfish skeletal muscles (Bradacs et al., 1997). In general, this phenomenon is readily explained by presynaptic calcium that accumulates even before the threshold for transmitter release is reached (Vyshedskiy and Lin, 1997). Across varying stimulus frequencies, even relatively simple forms of facilitation would be expected to show a threshold effect at low frequencies, maximal expression at a higher frequency, and potentially a decrease at even higher frequencies (Bayat Mokhtari et al., 2018). In the frequency range probed here (Fig. 5), this dynamic range was greater in *cpv2a* than *cpv2b*, as only *cpv2b* showed a sigmoidal dependence of responses across frequencies.

Differences in facilitation probed with paired-pulse and train stimulations (Figs. 4, 5), as well as differences in summation, gave some indication that repetitive input was integrated differently between *cpv2a* and *cpv2b*. However, the actual response dynamics must be evaluated during the type of inputs that the muscles usually receive, i.e., repetitive bursting. Repetitive bursting is a type of neuron and network activity found in many parts of the central nervous system, and burst frequency, spike frequency within bursts, and the way synaptic efficacy and resonance is shaped by short-term dynamic is an important aspect of neural coding in many contexts (Izhikevich et al., 2003; Zeldenrust et al., 2018). Of particular relevance here is the finding that the dynamics of *cpv2a* and *cpv2b* responses were not restricted to the temporal domain of single bursts but outlasted the cycle duration. Slow time constant augmentation of synaptic responses across bursts have been described in gastric mill stomach muscles (Stein et al., 2006), and have been shown to interact with short-term facilitation (Blitz et al., 2017). However, our results suggest that dynamics are more complex than can be explained by just two partially interacting processes.

One indication that synaptic dynamics was more complex than simple short-term facilitation was that response amplitudes did not just show a single exponential decay after a conditioning train (Fig. 4*C*,*D*). At both muscles, peak amplitudes did not occur at the smallest test pulse interval. In *cpv2a*, responses at larger intervals undershot the original amplitude, perhaps indicating a role of short-term depression, which can outlast short-term facilitation (Regehr, 2012). In *cpv2b*, responses recovered with two time constants, indicating that even at this small timescale facilitation can be shaped by several molecular processes (Vyshedskiy and Lin, 1997). Furthermore, episodic burst stimulations revealed that responses reached steady state only after several bursts, in a non-monotonic fashion that suggested the involvement of several processes acting at different timescales (Fig. 6). In *cpv2a*, steady-state responses were smaller than the initial one, again suggesting the involvement of depression. In *cpv2b*, steady-state responses were larger than the initial ones but less facilitated than after a few bursts, suggesting facilitation and/or depression at different timescales.

### Differential readout of input attributes during bursting

At steady state, responses to repetitive bursting revealed substantial differences in sensitivity to different input attributes (Fig. 7). In *cpv2a*, responses increased both with *F*_burst_ and *F*_spk_ or *Dur*, while *cpv2b* showed little sensitivity to changes in *F*_burst_. The latter is surprising in the light of the slow dynamics discussed above, but it could indicate a compensatory balance between several slower cellular processes under this stimulus regime, similar to the balance of short-term depression and facilitation at auditory brain stem synapses that can render response amplitudes fairly independent of input frequency (MacLeod et al., 2007). At both synapses, varying spike frequency and duration revealed bandpass filtering properties. While this is a common consequence of the presence of both facilitation and depression in many synapses (Markram et al., 1998), it plays a particularly significant role during rhythmic activation, giving rise to resonance phenomena (Izhikevich et al., 2003; Tseng et al., 2014).

In *cpv2a*, responses only decreased at *F*_burst_ > 1 Hz, which exceeds maximal values for pyloric rhythms in *Homarus* under control conditions *in vitro* (Bucher et al., 2005) and *in vivo* (Clemens et al., 1998). In *cpv2b*, responses were maximal at 0.6 Hz, meaning that this bandpass filtering property gave rise to a preferred frequency within the physiological range. Consequently, the relative amount of activation of *cpv2a* and *cpv2b* changed across rhythm frequencies. While changes in responses and resonance are readily explained by changes in *F*_spk_ in many cases (Izhikevich et al., 2003), a puzzling finding here was that *cpv2b* was more sensitive to changes in *Dur* than to changes in *F*_spk_ when the number of stimuli per burst was changed in a narrow range (Fig. 8). It is hard to speculate about underlying nonlinearities and processes with different time constants that could give rise to such a dependence, but it may be functionally significant when *Dur* and *F*_spk_ are controlled differentially. For example, *Dur* scales with cycle period across individuals, but *F*_spk_ does not (Bucher et al., 2005).

### Sensitivity to spike interval structure

Interspike interval signatures in pyloric neurons are shaped by synaptic dynamics (Szücs et al., 2003) and sensitive to central effects of neuromodulators (Szücs et al., 2005). In the PD neurons specifically, they can also be altered by spike propagation in a manner dependent on the presence of DA at the peripheral axons (Ballo and Bucher, 2009; Ballo et al., 2012). Whether muscle responses are sensitive to the fine temporal structure of bursts was unknown, and we show here that such changes are read out differentially between *cpv2a* and *cpv2b*. The inconsistent sensitivity to parabolic versus regularized burst inputs within each muscle (Fig. 9) is hard to reconcile with the rest of our results. Stomach muscle fibers within individual animals are homogenous, showing no indication of different fiber types within a single muscle (Govind and Lingle, 1987). Responses to current injections (Fig. 3) showed some qualitative differences between recordings, but only in *cpv2b*. Changes in interval structure that mimicked effects of spike propagation, however, were relatively consistent across individuals (Fig. 10). It is doubtful that the small changes in responses of *cpv2a* would have any functional significance for muscle activation, but the effects in *cpv2b* appeared substantial. Why such more subtle changes in input patterns have this consistent effect could be because the main changes were in the first intervals in each burst. As this change alters the burst duration, the effect could be related to the sensitivity of *cpv2b* to burst duration shown in Figure 8. However, in the experiments described in Figure 8, an 11% decrease in both *Dur* and number of spikes resulted in a 37% decrease in voltage responses. In contrast, duration varied only by ±2% across the stimulus patterns used in Figure 10. Alternatively, the effect may result from the dynamic of initial changes during the first interval in each burst, for example related to the time constants involved in the complex machinery that regulates presynaptic calcium dynamics (Regehr, 2012).

### Contributions of postsynaptic membrane nonlinearities

The contributions of postsynaptic membrane nonlinearities can vary substantially across different arthropod muscles. Skeletal muscles in crustaceans are commonly innervated by both phasic and tonic motor neurons. Phasic motor neurons often form depressing synapses on fast-twitching electrically excitable fibers, and tonic ones often form facilitating synapses on relatively unexcitable slowly contracting fibers (Atwood, 2002, 2008). Even excitable fibers mostly do not produce full-blown all-or-none action potentials, but membrane nonlinearities generate smaller voltage-dependent potentials or GAPs that amplify synaptic inputs (Atwood, 1963, 2002). Full spiking ability is masked by substantial voltage-gated and/or Ca^2+^-gated K^+^ currents (Araque and Buño, 1995; Araque et al., 1998; Weiss et al., 2001).

In contrast to limb and abdominal muscles, most crustacean stomach muscles exclusively comprise slow and unexcitable fibers (Govind and Lingle, 1987). While a few stomach muscles can show highly nonlinear membrane properties like spontaneous oscillatory and spiking behavior (Lingle, 1981; Meyrand and Moulins, 1986; Meyrand, 1987; Meyrand and Marder, 1991), typical GAPs have previously only been shown in embryonic and larval preparations (Richards et al., 2003). Both our observation of GAPs (Fig. 2) and the subthreshold nonlinearities revealed by current injections (Fig. 3) suggest that muscle fiber membrane properties could contribute substantially to the different sensitivities to input pattern attributes in *cpv2a* and *cpv2b*. The rarity of GAPs and the dampening of depolarizing inputs observed in *cpv2a* may mean that intrinsic membrane properties are counteracting the effects of synaptic facilitation. In contrast, the higher excitability and amplification of depolarizing inputs in *cpv2b* may enhance the effects of synaptic facilitation.

### Functional consequences for motor output

Ultimately, motor behavior arises from the movement of joints and body parts produced by muscle contractions, and muscle fiber synaptic responses are only an intermediate step in the highly nonlinear relationship between motor neuron spike pattern and movement production, the neuromuscular transform (Brezina et al., 2000; Williams et al., 2013). Intracellular calcium transients and subsequent contractions likely have a complex dependence on EJP amplitude, voltage integral, and history of activation (Kuo and Ehrlich, 2015). While we assume that contraction responses are sensitive to the differences in electrical responses described here, how the sensitivity to input pattern changes compare between electrical and contraction responses is hard to predict.

The biomechanics of the pyloric filter apparatus are barely understood, as movements are generated by a complex set of muscles attached to a large number of ossicles (Maynard and Dando, 1974). The two muscles under investigation here are both ventral dilators which run in parallel for most of their length and have close insertion sites at the pylorus. This arrangement is reminiscent of other largely parallel muscles with differences in physiology and activation, like the human gastrocnemius and soleus calf muscles (Herman, 1967; Alrowayeh et al., 2011), but those muscles are innervated by separate groups of lower motor neurons. In contrast, *cpv2a* and *cpv2b* receive the same PD neuron input, but read out this input differently. The electrical responses suggest that *cpv2b* activation lags behind *cpv2a* (Figs. 1*D*, 5*C*), and that the relative amount of activation between the two muscles changes with input patterns. Stomach muscle contractions are slow and summate across bursts, resulting in phasic contractions that ride on top of a tonic baseline tension (Morris and Hooper, 1998; Hooper and Weaver, 2000). The relative expression of phasic versus tonic contractions is sensitive to different input attributes (Morris and Hooper, 1997, 1998), which could be an important component of differential readout between *cpv2a* and *cpv2b*.

Electrical and contraction responses of crustacean stomach muscles do not just depend on input patterns but are also altered by neuromodulators. Neuromodulators affect circuit output and thus indirectly change muscle outputs, but hemolymph-borne modulators such as DA reach the muscles and thus can also have a direct effect. These neurohormones can change neuromuscular synaptic efficacy and dynamics, as well as contraction properties (Govind and Lingle, 1987; Jorge-Rivera and Marder, 1997; Jorge-Rivera et al., 1998). Therefore, both the motor commands and their readout can be dynamically adjusted, and modulators could alter the sensitivity to different attributes of input patterns in both muscles.

## Synthesis

Reviewing Editor: Arianna Maffei, SUNY Stony Brook Decisions are customarily a result of the Reviewing Editor and the peer reviewers coming together and discussing their recommendations until a consensus is reached. When revisions are invited, a fact-based synthesis statement explaining their decision and outlining what is needed to prepare a revision will be listed below. The following reviewer(s) agreed to reveal their identity: Ronald Calabrese, Patsy Dickinson.

There is consensus between the reviewers that the manuscript provides a valuable contribution to the field.

The reviewers agreed that they do not have major concerns.

Their specific minor points are provided below.

Reviewer #1

In this work, the authors explore in detail postsynaptic response dynamics at two pyloric muscles in the lobster foregut innervated by the same pair of PD motor neurons. They show that the muscles differ fundamentally in their postsynaptic response dynamics to motor neuron input patterns both artificial and similar to those during in vivo rhythmic activity. By cleverly manipulating the input patterns they disambiguate the effects of burst period, burst duration and intraburst spike frequency. They also explore how very subtle changes in the initial spike interval of a burst affects the postsynaptic response dynamics, such as those that can be induced by DA modulation of spike transmission in the PD motor neurons. The techniques employed are standard electrophysiology and the experiments are carefully performed and thoroughly analyzed and scrutinized with appropriate statistics.

The bottom line is that the experiments are well performed, and a rich data set is generated.

Revisions

1. A concern is that activation of the PD motor neuron by pdn extracellular stimulation, likely activates both PD motor neurons - no mention of looking for a distinct threshold is made - and thus represents a synchronous activation that could enhance the effect of the subtle spike arrivals that are represented in the stimulation patterns of Figure 10. In vivo the two PD spikes will not arrive at the same time making this initial interval more complex than the canonical pattern used in these experiments where two sets of synapses are activated together and thus ejps augmented. Thus, the functional differences of this subtle time difference in vivo, where the two sets of synapses are asynchronously activated, may not be resolved by these experiments. Please discuss.

2. Because, as the authors explicitly recognize, their current clamp recordings assess the combined effects of synaptic dynamics (due to postsynaptic currents) interacting with postsynaptic membrane dynamics it is inappropriate to call these ‘synaptic dynamics’. Postsynaptic response dynamics would be more accurate.

3. Line 173: Stomach muscle fibers are multiterminally innervated along their length, which ensures spatial spread of... As written, it implies design.

4. Lines 261, 262, and 591; is temper(ing) the right word here? Dampen(ing)?.

5. Lines 584-588: I could not follow the logic here.

6. Lines 425-426 “Blocking Ih exacerbates pattern changes, while DA-mediated increase in Ih alleviates them (Ballo et al., 2012; Zhang et al., 2017). Please explain more fully.

7. In general the Discussion seems too focused on synaptic dynamics - explained above that this should mean only the dynamics of postsynaptic currents reflecting presynaptic release - and not enough focused on the functional implications of the study. I am not sure this study contributes much new to our understanding of synaptic dynamics.

Reviewer #2

My comments, given by line number, are all quite minor, but should improve the readability of the manuscript:

Line 102: Wording of first part of line is not clear - too many stacked modifiers.

Line 107: “were” should be “where"

Lines 120-121: the last phrase is essentially an incomplete sentence; please re-word.

Line 154: “Regression analyses was” should be either ‘analyses were’ or ‘analysis was’

Lines 193-194 and Fig 1D: To me it seems like part of the reason for the relatively smooth appearance of the peaks marked by the white arrow is that they are earlier In the train and therefor are much smaller than the later ones, presumably due to the differences in facilitation, not necessarily due to differences in arrival times of the PD neurons. It seems like this should be mentioned as part of the explanation.

Lines 201-2: I assume the recordings here are in parts of the muscle that are equidistant from the STG in the two muscles, but it would be nice to state that explicitly.

Line 211: Are the numbers here (202 and 216) the number of animals recorded from for each of the muscles? It would be helpful to clarify (perhaps in Methods) since that is a larger number than for most reports of this type. (And if it is not, please note what they actually represent.)

Lines 238-242: Aren’t these actually (technically) V-I curves rather than I-V since they are done in current clamp?

Line 248: Please include a reference to the use of TEA for blocking Ca-gated K channels (here or in Methods).

Line 250: Consider adding ’TTX-sensitive” before voltage-gated Na channels’

Line 259 and Figure 3: Although the number of experiments using TEA is given for cpv2a, no data are shown. How do these recordings compare to those shown for cpv2b? I would really like to see those data as well.

Lines 315-317: Please explain this more clearly

Lines 395-7 and Figure 8: Is it possible to label the figure more completely (especially part B graphs)? I generally found this figure and the explanation a bit difficult to parse; the differences in the color tones are quite small on my screen, and trying to put the details of the graphs together with the figures was not immediately evident. Moreover, since this is mostly a color-coded figure, having the teal mean several different things (eg in the graphs of part A vs the arrowheads in part B) doesn’t really help. The data are nice - but if it could be made easier for the reader to interpret, that would be helpful.

Figure 3B: Why not just use current values rather than step number? That way the reader wouldn’t have to do the calculations to know what the x-axis values are.
